# Computational Models for Calcium-Mediated Astrocyte Functions

**DOI:** 10.3389/fncom.2018.00014

**Published:** 2018-04-04

**Authors:** Tiina Manninen, Riikka Havela, Marja-Leena Linne

**Affiliations:** Computational Neuroscience Group, BioMediTech Institute and Faculty of Biomedical Sciences and Engineering, Tampere University of Technology, Tampere, Finland

**Keywords:** astrocyte, astrocyte network, computational model, glia, intracellular calcium, neuron-astrocyte network, simulation, synapse

## Abstract

The computational neuroscience field has heavily concentrated on the modeling of neuronal functions, largely ignoring other brain cells, including one type of glial cell, the astrocytes. Despite the short history of modeling astrocytic functions, we were delighted about the hundreds of models developed so far to study the role of astrocytes, most often in calcium dynamics, synchronization, information transfer, and plasticity *in vitro*, but also in vascular events, hyperexcitability, and homeostasis. Our goal here is to present the state-of-the-art in computational modeling of astrocytes in order to facilitate better understanding of the functions and dynamics of astrocytes in the brain. Due to the large number of models, we concentrated on a hundred models that include biophysical descriptions for calcium signaling and dynamics in astrocytes. We categorized the models into four groups: single astrocyte models, astrocyte network models, neuron-astrocyte synapse models, and neuron-astrocyte network models to ease their use in future modeling projects. We characterized the models based on which earlier models were used for building the models and which type of biological entities were described in the astrocyte models. Features of the models were compared and contrasted so that similarities and differences were more readily apparent. We discovered that most of the models were basically generated from a small set of previously published models with small variations. However, neither citations to all the previous models with similar core structure nor explanations of what was built on top of the previous models were provided, which made it possible, in some cases, to have the same models published several times without an explicit intention to make new predictions about the roles of astrocytes in brain functions. Furthermore, only a few of the models are available online which makes it difficult to reproduce the simulation results and further develop the models. Thus, we would like to emphasize that only via reproducible research are we able to build better computational models for astrocytes, which truly advance science. Our study is the first to characterize in detail the biophysical and biochemical mechanisms that have been modeled for astrocytes.

## 1. Introduction

Astrocytes have traditionally been regarded as glial cells responsible for the homeostasis and metabolic support for neurons (Carmignoto and Gómez-Gonzalo, 2010). Current evidence indicates that astrocytes support neuronal functions also in many other ways. Astrocytes are in a close proximity to a large number of brain synapses (Bushong et al., 2002; Fellin et al., 2006; Halassa et al., 2007; Oberheim et al., 2009; Freeman, 2010; Volterra et al., 2014; Olude et al., 2015; Cali et al., 2016), and the astrocytes together with pre- and postsynaptic neurons have been proposed to form functional tripartite synapses (Araque et al., 1999). Astrocytes can thus broadly sense and regulate synaptic activity. A growing body of evidence also suggests that astrocytes modulate neural circuits and are involved in many brain functions, perhaps also in cognitive functions and behavior (Perea et al., 2009; Wang et al., 2009; Halassa and Haydon, 2010; Henneberger et al., 2010; Suzuki et al., 2011; Araque et al., 2014; Sibille et al., 2014; Khakh and Sofroniew, 2015; Poskanzer and Yuste, 2016). Although more and more evidence is accumulating on the astrocytic modulation of neurotransmission and synaptic plasticity, *in vivo* evidence to support that astrocytes are directly activated by neurotransmission and signal back to neurons to modulate neurons' output remains unclear. Particularly, how this modulation occurs in time and space is unresolved.

Since the 1990s a variety of experimental techniques and organisms have been used to study astrocytes. Until 2010 most of the studies were performed using *in vitro* cell cultures and slice preparations. Recently, studies addressing astrocytes' roles in brain functions *in vivo* have accumulated. In short, one could identify three waves of astrocyte research over the past three decades, as proposed by Bazargani and Attwell (2016). The first wave of evidence revealed that neurotransmitter glutamate increases the astrocytic calcium (Ca^2+^) concentration *in vitro* and this yields to Ca^2+^ wave propagation between astrocytes (Cornell-Bell et al., 1990; Charles et al., 1991; Dani et al., 1992; Newman and Zahs, 1997), which could lead to Ca^2+^ increase in the nearby neurons (Nedergaard, 1994; Parpura et al., 1994). The second wave of evidence showed that pharmacological tools used to separate astrocytic and neuronal components are not selective (Parri et al., 2001; Agulhon et al., 2010; Hamilton and Attwell, 2010). Furthermore, it was speculated that astrocytic processes close to synapses do not have endoplasmic reticulum (ER) present and that blocking the inositol trisphosphate (IP_3_) receptors (IP_3_Rs) in the astrocytes has an effect on the astrocytic Ca^2+^ but not on the synaptic events (Fiacco et al., 2007; Petravicz et al., 2008; Agulhon et al., 2010; Patrushev et al., 2013). The third wave of evidence (Bazargani and Attwell, 2016) led to the conclusion that the Ca^2+^ transients in the astrocytic processes near vascular capillaries (Otsu et al., 2015) and neuronal synapses (Nimmerjahn et al., 2009) and not in the soma are the key that needs to be addressed in more detail. In summary, the challenges in astrocyte research have been the lack of selective pharmacological tools and the partially contradictory results obtained in *in vivo* in contrast to various *in vitro* preparations.

Although there is partial controversy, which hinders attempts to explain all findings on astrocytes' roles in the central nervous system in an unambiguous way, the majority of data collected over the past decades strongly suggests that fluctuations in Ca^2+^ concentrations in both soma and processes are important measures of astrocytic activities. Then astrocytic Ca^2+^ activity is utilized, in one way or another, by neurons to sense ongoing neural activity in closeby or more distant networks. The dynamic, far-reaching fluctuations, or transients, in astrocytic Ca^2+^ concentration were also recently recorded in awake behaving mice *in vivo* by several independent studies (Ding et al., 2013; Paukert et al., 2014; Srinivasan et al., 2015). Moreover, astrocytes, similarly to any other cell in the mammalian body, are known to express an overwhelming complexity of molecular and cell-level signaling. The full complexity of the signaling pathways which control Ca^2+^ transients and exert their effects in astrocytes is poorly understood, and the question about their relevance in awake behaving animals remains unanswered. It is essential that the research community seeks to systematically characterize the key signaling mechanisms in astrocytes to understand the interactions between different systems, including neuronal, glial, and vascular, in brain circuitry. Astrocytic signaling may provide a potential, widespread mechanism for regulating brain functions and states (Yang et al., 2014; Haim and Rowitch, 2017).

Several factors might be important in orchestrating how astrocytes exert their functional consequences in the brain. These include (a) different receptors or other mechanisms that trigger an increase in Ca^2+^ concentration in astrocytes, (b) Ca^2+^-dependent signaling pathways or other mechanisms that govern the production and release of different mediators from astrocytes, and (c) released substances that target other glial cells, the vascular system, and the neuronal system. The listed three factors (a–c) operate at different temporal and spatial scales and depend on the developmental stage of an animal and on the location of astrocytes. Namely, a substantial amount of data on a diverse array of receptors to detect neuromodulatory substances in astrocytes *in vitro* has been gathered (Backus et al., 1989; Kimelberg, 1995; Jalonen et al., 1997), and accumulating evidence is becoming available for *in vivo* organisms as well (Beltrán-Castillo et al., 2017). Neuromodulators have previously been expected to act directly on neurons to alter neural activity and animal behavior. It is, however, possible that at least part of the neuromodulation is directed through astrocytes, thus contributing to the global effects of neurotransmitters (see e.g., Ma et al., 2016). Experimental manipulation of astrocytic Ca^2+^ concentration is not a straightforward practice and can produce different results depending on the approach and context (for more detailed discussion, see e.g., Agulhon et al., 2010; Fujita et al., 2014; Sloan and Barres, 2014). Additional tools, both experimental and computational, are required to understand the vast complexity of astrocytic Ca^2+^ signaling and how it is decoded to advance functional consequences in the brain.

Several reviews of theoretical and computational models have already been presented (for a review, see e.g., Jolivet et al., 2010; Mangia et al., 2011; De Pittà et al., 2012; Fellin et al., 2012; Min et al., 2012; Volman et al., 2012; Wade et al., 2013; Linne and Jalonen, 2014; Tewari and Parpura, 2014; De Pittà et al., 2016; Manninen et al., 2018). We found out in our previous study (Manninen et al., 2018) that most astrocyte models are based on the models by De Young and Keizer (1992), Li and Rinzel (1994), and Höfer et al. (2002), of which the model by Höfer et al. (2002) is the only one built specifically to describe astrocytic functions and data obtained from astrocytes. Some of the other computational astrocyte models that steered the field are the models by Nadkarni and Jung (2003), Bennett et al. (2005), Volman et al. (2007), De Pittà et al. (2009a), Postnov et al. (2009), and Lallouette et al. (2014). However, irreproducible science, as we have reported in our other studies, is a considerable problem also among the developers of the astrocyte models (Manninen et al., 2017, 2018; Rougier et al., 2017). Several other review, opinion, and commentary articles have addressed the same issue as well (see e.g., Cannon et al., 2007; De Schutter, 2008; Nordlie et al., 2009; Crook et al., 2013; Topalidou et al., 2015; McDougal et al., 2016). We believe that only via reproducible science are we able to build better computational models for astrocytes and truly advance science.

This study presents an overview of computational models for astrocytic functions. We only cover the models that describe astrocytic Ca^2+^ signaling by biophysical means. We first categorize the existing models based on whether they are describing astrocytes or neuron-astrocyte interactions. We have previously described some aspects of the astrocyte and neuron-astrocyte models in our related study (Manninen et al., 2018), where we listed the details of both the astrocyte and neuron models in a simplistic, educational manner. In this review, we characterize in detail the existing models based on what kind of astrocytic mechanisms have been taken into account. Our study is expected to help guide future computational studies addressing the cross-talk between astrocytes and other systems in the brain and help researchers select suitable models for their research questions.

## 2. Materials and methods

In this section, we first outline the basics of astrocyte biology. The mechanisms presented here are not typically included in computational neuroscience models and one of our aims is to carefully assess which of the mechanisms are presently modeled and how realistically. We then list the computational astrocyte models, developed by the end of 2017, for our detailed evaluation. At the end of the section, we give the details how we characterized the models.

### 2.1. Astrocyte biophysics and biochemistry for modeling of astroglial functions

Astrocytes are the most diverse glial cells in the central nervous system. Astrocytes from different brain regions differ in morphology, physiology, and expression of genes encoding the most fundamental proteins responsible for astroglial function. In general, astrocytes can have a soma, perisynaptic processes which engulf neuronal synapses and also enclose some extracellular space, called perisynaptic or extrasynaptic (or sometimes periastrocytic) space within, and perivascular processes which connect the astrocyte with blood vessels and enclose some extracellular space called perivascular space. Below we present a generic view of some of the most important biophysical and cellular mechanisms that are shown to underlie important astrocytic functions (for more information, see also Kettenmann and Ransom, 2013; Verkhratsky and Butt, 2013).

#### 2.1.1. Ion distribution and ion channels for basic membrane excitability

Astroglial cells express all major ion channel types, including potassium (K^+^), sodium (Na^+^), and Ca^2+^ channels, and also various types of anion and chloride (Cl^−^) channels, water channels (aquaporins), transient receptor potential (TRP) channels, and non-selective channels. The ion distribution is also somewhat different from neurons: intracellular concentrations of K^+^ and Ca^2+^ are similar to neurons, but the concentrations of Na^+^ and especially Cl^−^ are higher compared to neurons. Astrocytes have a rather negative resting membrane potential (around −80 to −90 mV) because of the predominance of K^+^ conductance. Electrical depolarization of astroglia does not produce regenerative action potentials as in neurons.

Ca^2+^-mediated signals have been proposed to be the main mediator of communication between astrocytes and other cellular elements in the brain (Nimmerjahn, 2009; Volterra et al., 2014; Bazargani and Attwell, 2016). Transient Ca^2+^ increases restricted to single cells are called Ca^2+^ oscillations. In isolated astrocytes, intracellular Ca^2+^ oscillations have been shown to depend mainly on the Ca^2+^-induced Ca^2+^ release (CICR) via IP_3_Rs from the ER to the cytosol (see e.g., Agulhon et al., 2008). CICR has been shown to depend on Ca^2+^ with or without the influence of IP_3_. Ca^2+^ influx via voltage-gated Ca^2+^ channels (VGCCs) and other types of Ca^2+^ influxes from the extracellular space have been linked with Ca^2+^ oscillations as well (Aguado et al., 2002).

#### 2.1.2. Membrane transporters for uptake and homeostatic control of ions, neurotransmitters, and other substances

The membrane transporters are particularly important for astroglia because they control movements of various substances, including ions, neurotransmitters, and metabolic substrates. Astroglial transporters include adenosine and adenosine triphosphate (ATP)-dependent transporters such as the Na^+^/K^+^-ATPase (NKA, also called Na^+^/K^+^ pump) and Ca^2+^-ATPase [also called Ca^2+^ pump or plasma membrane Ca^2+^-ATPase (PMCA)] on the plasma membrane, in addition to sarco/ER Ca^2+^-ATPase (SERCA) located on the ER membrane. They also contain so-called secondary transporters, such as glutamate transporters [excitatory amino acid transporters (EAATs)], gamma-aminobutyric acid (GABA) transporters, glycine transporters, Na^+^/Ca^2+^ exchangers (NCXs), Na^+^/hydrogen (H^+^) exchangers, Na^+^/bicarbonate (${\text{HCO}}_{3}^{-}$) cotransporters, Na^+^/K^+^/Cl^−^ cotransporters (NKCC1), and some others. Although, for example, glutamate transporters are expressed by all cell types in the brain, astrocytes are the main cell type responsible for glutamate uptake. Astrocytes have enzymes that convert both glutamate and GABA into glutamine. Glutamine is then released into the extracellular space and taken up by the presynaptic terminal, and can be converted to glutamate or GABA.

The NKCC1 cotransporter specifically contributes to the regulation of extracellular K^+^ homeostasis in the central nervous system. During excessive neuronal firing, the local extracellular K^+^ concentration can increase markedly and lead to neural dysfunction. Uptaking extracellular K^+^, transporting it intracellularly and releasing to areas with lower concentration are some of the most important functions of astrocytes. This process of intracellularly transporting K^+^ from extracellular areas of high concentration to areas of low concentration, via active uptake and release, is called spatial K^+^ buffering. K^+^ can also diffuse in extracellular space which also helps in lowering the local high concentration. Furthermore, astrocytes also buffer other ions than K^+^.

#### 2.1.3. Ionotropic and metabotropic receptors for sensing the environment

Astrocytes are able to sense with the help of their receptors local and more distant environments, including the neural activity and the synaptically released neurotransmitters, both in *in vitro* cell cultures and brain slices as well as in *in vivo* (Glaum et al., 1990; Dani et al., 1992; Porter and McCarthy, 1996; Hirase et al., 2004). Astroglial cells can express the same variety of receptors as neurons, both ionotropic and metabotropic. These include glutamate, GABA, glycine, acetylcholine, adrenergic, serotonin, histamine, cannabinoid, and neuropeptide receptors, and purinoceptors for adenosine and ATP. For example, extracellular ATP can bind to astroglial purinergic G-protein receptors, resulting in IP_3_-mediated CICR, and, eventually, release of ATP to the extracellular space.

#### 2.1.4. Release of transmitters and modulators

As described above, astroglia are capable of receiving signals from neurons via membrane receptors and converting the received information into Ca^2+^ excitability. The fact that astrocytes could release extracellular signaling molecules, regulated by this Ca^2+^ excitability, not only to the vascular system but also to the neuronal one implies a very active role for astroglia in the brain. The concept of regulated transmitter/modulator release from astrocytes to neurons is generally known as gliotransmission (Parpura et al., 1994; Bezzi and Volterra, 2001), and the released substances are referred to as gliotransmitters. Most common gliotransmitters are glutamate, D-serine, and ATP (Parpura et al., 1994; Parri et al., 2001). Astrocytes are, in general, thought to release transmitters, and probably also modulators, by several different mechanisms, which can be broadly divided into exocytotic release, diffusional release through membrane pores, and transporter mediated release. It is, however, not known which of these mechanisms are used in certain astroglial functions.

These terms “gliotransmission” and “gliotransmitter” may be somewhat misleading as the very same transmitters are also released by neurons. Moreover, it is assumed that a mechanism similar to vesicular release from neuronal synaptic terminals exists also in astrocytes. Some studies have indeed detected vesicle-type structures in astrocytes *in vitro*, and thus it has been proposed that gliotransmitter release might be purely vesicular. It is, however, important to bear in mind that the existence of molecular machinery for packing gliotransmitters into vesicles and for arranging the vesicles into a readily releasable pool has not so far been supported by experimental evidence *in vivo* (see e.g., Fujita et al., 2014; Sloan and Barres, 2014). More evidence on the release mechanism, using improved experimental model systems and techniques that allow studies at deeper resolution in physiological conditions, is required (Li et al., 2013; Bazargani and Attwell, 2016; Fiacco and McCarthy, 2018; Savtchouk and Volterra, 2018).

In our evaluation of models, we use the term “gliotransmission” for all biophysical and phenomenological mechanisms that were modeled to take into account the release of substances from astrocytes and targeting neurons. The reason for this is that the term “gliotransmission” is often used in the original modeling publications.

In addition, glutamate released from astrocytes can activate extrasynaptic N-methyl-D-aspartate receptor (NMDAR)-dependent currents, often called NMDAR-dependent slow inward current (SIC). In modeling studies, SIC is many times modeled similarly to, for example, the modulating current (*I*_astro_) presented by Nadkarni and Jung (2003).

#### 2.1.5. Connexin-based gap junction hemichannels

It is not just neurons that form networks but also astrocytes. A fundamental difference between neuronal and astroglial networks is that astrocytes are connected, through gap junctions composed mainly of connexin 43 hemichannels, to form a functional cellular syncytium in the central nervous system. In their open state, these channels are permeable to large hydrophilic solutes with molecular mass of several hundred Daltons, and are permeable to small solutes in their closed state (see e.g., Bao et al., 2007). The gap junction connectivity is instrumental for astrocytes' functions, including generation of Ca^2+^ waves, water transport, K^+^ buffering, and control of vascular system, and is one of the most important mechanisms to be modeled in astrocyte networks.

Three different pathways have been discovered so far to induce Ca^2+^ waves in astroglial networks. The first route depends on the transfer of IP_3_ via gap junctions (Giaume and Venance, 1998). Transported IP_3_ via gap junctions triggers CICR in the coupled astrocytes and induces Ca^2+^ wave propagation in astroglial syncytium. The second route to induce Ca^2+^ waves depends on the extracellular diffusion of ATP (see e.g., Newman and Zahs, 1997; Guthrie et al., 1999 and section 2.1.4). The third route has been shown to depend on extracellularly applied potassium chloride, causing, among others, a pathophysiological phenomenon called cortical spreading depression (Peters et al., 2003). The regulation of gap junction communication within the astroglial syncytium is a complex process and is intensively studied.

Most of the above described biophysical and biochemical mechanisms have been modeled in some detail in astrocytes. Below we address altogether 106 models developed until the end of 2017 and describe their capacity to represent the dynamics of astrocyte biophysics and biochemistry.

### 2.2. Selection of models

The number of computational astrocyte models has increased over the past couple of years. This increase in the number of models does not, however, solely reflect the vast amount of experimental data that is currently presented for astrocytes and for their roles in brain functions and the regulation of the neuronal system. Several focused reviews of computational astrocyte and neuron-astrocyte models have appeared during the last few years (see e.g., Jolivet et al., 2010; Mangia et al., 2011; De Pittà et al., 2012; Fellin et al., 2012; Min et al., 2012; Volman et al., 2012; Wade et al., 2013; Linne and Jalonen, 2014; Tewari and Parpura, 2014; De Pittà et al., 2016; Manninen et al., 2018); of which our study (Manninen et al., 2018) is the most comprehensive evaluation of more than 60 models published by the end of 2014. In the present study, we characterize in more detail the biophysical and biochemical components of astrocytes that were taken into account in the astrocyte and neuron-astrocyte interaction models published by the end of 2017.

Table 1 presents altogether 106 astrocyte models. As in our other study (Manninen et al., 2018), we here limited our evaluation of models to astrocytic signal transduction pathways that were defined using several characteristics. First, models were able to include pre- and postsynaptic neuron models as part of the whole models. Second, part of intracellular signaling in the astrocytes was explicitly modeled, thus models were required to include (biophysical) mechanisms for astrocytic Ca^2+^ dynamics. We considered in the present study only models where astrocytic Ca^2+^ signaling was described by a differential equation that was a function of time and at least one of the other astrocytic variables, for example IP_3_. Third, astrocytic Ca^2+^ affected some signaling variables or other intracellular signals in the astrocytes. Models which described Ca^2+^ dynamics but were not explicitly made for astrocytes were excluded from the present study. Moreover, models that mainly concentrated on describing ionic homeostasis, such as regulation of extracellular K^+^ ions, were also excluded from the evaluation unless they incorporated astrocytic Ca^2+^ signaling. These strict criteria were needed because of the large number of models.

**Table 1:** List of astrocyte and neuron-astrocyte models published each year.

**Year**	**Models**	**No**.
1995	Roth et al., 1995	1
2002	Höfer et al., 2002	1
2003	Nadkarni and Jung, 2003	1
2004	Goto et al., 2004; Nadkarni and Jung, 2004	2
2005	Bellinger, 2005; Bennett et al., 2005; Larter and Craig, 2005; Nadkarni and Jung, 2005	4
2006	Bennett et al., 2006; Iacobas et al., 2006; Stamatakis and Mantzaris, 2006; Ullah et al., 2006	4
2007	Di Garbo et al., 2007; Gibson et al., 2007; Nadkarni and Jung, 2007; Postnov et al., 2007; Stamatakis and Mantzaris, 2007; Volman et al., 2007	6
2008	Bennett et al., 2008a,b; Gibson et al., 2008; Lavrentovich and Hemkin, 2008; Nadkarni et al., 2008; Silchenko and Tass, 2008	6
2009	Allegrini et al., 2009; De Pittà et al., 2009a,b; Di Garbo, 2009; Kang and Othmer, 2009; Kazantsev, 2009; Postnov et al., 2009; Zeng et al., 2009	8
2010	Edwards and Gibson, 2010; Ghosh et al., 2010; Goldberg et al., 2010; Skupin et al., 2010; Sotero and Martínez-Cancino, 2010	5
2011	Amiri et al., 2011a,b; DiNuzzo et al., 2011; Dupont et al., 2011; Farr and David, 2011; Matrosov and Kazantsev, 2011; Riera et al., 2011a,b; Toivari et al., 2011; Valenza et al., 2011; Wade et al., 2011; Wei and Shuai, 2011	12
2012	Amiri et al., 2012a,b,c; Chander and Chakravarthy, 2012; Li et al., 2012; Tewari and Majumdar, 2012a,b; Wade et al., 2012; Witthoft and Karniadakis, 2012	9
2013	Amiri et al., 2013a,b; Diekman et al., 2013; Hadfield et al., 2013; Liu and Li, 2013a,b; MacDonald and Silva, 2013; Tang et al., 2013; Tewari and Parpura, 2013; Witthoft et al., 2013	10
2014	Lallouette et al., 2014; López-Caamal et al., 2014; Montaseri and Yazdanpanah, 2014; Wallach et al., 2014	4
2015	Komin et al., 2015; Mesiti et al., 2015a,b; Naeem et al., 2015; Nazari et al., 2015a,b,c; Soleimani et al., 2015; Yang and Yeo, 2015	9
2016	Amiri et al., 2016; De Pittà and Brunel, 2016; Haghiri et al., 2016; Hayati et al., 2016; Li et al., 2016a,b,c; Liu et al., 2016; Oku et al., 2016; Tang et al., 2016	10
2017	Chan et al., 2017; Guo et al., 2017; Haghiri et al., 2017; Handy et al., 2017; Li et al., 2017; Nazari et al., 2017; Oschmann et al., 2017; Taheri et al., 2017; Tang et al., 2017	9
2018	Ding et al., 2018; Karimi et al., 2018; Kenny et al., 2018; Liu et al., 2018; Yao et al., 2018	5
All		106

*Models are ordered alphabetically for each year of publication. Altogether 106 models have been published by the end of 2017. For chosen criteria, see section 2.2*.

### 2.3. Characteristics of models

We first categorized and tabulated the existing models based on whether they were describing single astrocytes, astrocyte networks, neuron-astrocyte synapses, or neuron-astrocyte networks. Next, we categorized the models further to see which older models were used as references. Most of the astrocyte models utilized the Ca^2+^ dynamics models by De Young and Keizer (1992) and Li and Rinzel (1994), or a modification of them, even though these two models were not made to describe astrocytic behavior (Manninen et al., 2018). Thus, the details of these models (De Young and Keizer, 1992; Li and Rinzel, 1994) were not listed. Models are in alphabetical order, first are models that used the models by De Young and Keizer (1992) and Li and Rinzel (1994) as references, second are the models that used the model by Höfer et al. (2002) as reference, third are the models that combined all three models (De Young and Keizer, 1992; Li and Rinzel, 1994; Höfer et al., 2002), and last are models that were based on some other models, such as the models by Sneyd et al. (1994), Keener and Sneyd (1998, 2009), and Houart et al. (1999) (see section 3). It is worth noting that most of the above-mentioned models have adapted CICR model presented by Bezprozvanny et al. (1991) as basis of their models.

Next, we characterized the models based on what type of signaling events were modeled in astrocytes. We listed for each model the number of astrocytes modeled, input used for the astrocytic module, astrocytic variables described by differential equations, and astrocytic Ca^2+^ fluxes related to cytosolic Ca^2+^. It is important to note that neurotransmitters were not named here as astrocytic variables but in some models they were used as inputs to the astrocyte models [e.g., synaptic glutamate ([Glu]_syn_) or neurotransmitter (NT)]. By contrast, we listed gliotransmitters as astrocytic variables if they were modeled with a differential equation. As the exact mechanisms of astrocytic signaling that regulate exocytosis and release of molecular substances from astrocytes are still largely unknown, most of the models used phenomenological descriptions of gliotransmitters or other substances released. Proposed gliotransmitters included, for example, glutamate ([Glu]_ext_) and ATP ([ATP]_ext_). Note the difference between [Glu]_syn_ and [Glu]_ext_, the former meaning the neurotransmitter glutamate released from the presynaptic neuron and the latter meaning the gliotransmitter glutamate released from the astrocyte. Other examples of strategies for modeling gliotransmission or similar exocytotic or modulating signaling mechanisms from astrocytes to neurons included modeling currents depending on astrocytic Ca^2+^ (*I*_astro_, Nadkarni and Jung, 2003) and several other types of currents depending, for example, on astrocytic mediator (*G*_m_, *I*_ast_, Postnov et al., 2007, 2009), or modeling phenomenological gating variable (*f*, Volman et al., 2007). In addition, we listed diffusion of astrocytic variables either in the cytosol, ER, or extracellular space. In the present study, we did not catalog the transfer of molecules or ions between intracellular space and extracellular space under the attribute “diffusion,” but we listed, for example, different Ca^2+^ related fluxes over the plasma membrane under “Ca^2+^ fluxes.” We chose to list gap junction signaling between astrocytes which is an important, fundamental form of intercellular signaling directly from the intracellular space of one cell to the intracellular space of another cell. We did not incorporate gap junctions under the attribute “diffusion,” but only included it in the attribute “gap junction.” IP_3_ and Ca^2+^ are examples of second messengers that are able to pass through gap junctions. The last items we listed were the output of the astrocytic module and experimentally shown event that the model was finetuned to capture [Ca^2+^ dynamics (Ca^2+^), homeostasis (Home.), vascular (Vasc.), synchronization (Synch.), information transfer (Inf.), plasticity (Plast.), and hyperexcitability (Hyper.)] for each model. To get a general idea of how these different mechanisms can be modeled, see, for example, books by Keener and Sneyd (2009) and Dupont et al. (2016).

Sometimes the authors of the publications used incorrect terminology when explaining their model components. As an example, Silchenko and Tass (2008), Zeng et al. (2009), Sotero and Martínez-Cancino (2010), Dupont et al. (2011), Li et al. (2012), and Ding et al. (2018) presented one Ca^2+^ flux as a leak from cytosol into extracellular space in their study. We named it here as Ca^2+^ efflux because Ca^2+^ leak flux via astrocytic cell membrane is normally used for a flux from extracellular space into cytosol, thus from the larger Ca^2+^ concentration in the extracellular space toward the smaller Ca^2+^ concentration in the cytosol. Goto et al. (2004) named the direction of fluxes incorrectly for CICR, ER leak flux, and ER pump (that we assumed to be SERCA) in their text, but in their equations the direction of fluxes were marked correctly for CICR and leak and again incorrectly for ER pump. Yao et al. (2018) also marked the signs incorrectly for some of the fluxes. Directions of the CICR and ER leak flux should be from the ER to the cytosol, and SERCA pump from the cytosol to the ER. Often the authors of the modeling publications did not name their Ca^2+^ pump on the membrane of ER as SERCA pump, but since the very same equation was used for SERCA pump by other authors, we marked it as SERCA pump for all the authors, except for the model by Guo et al. (2017) because they pointed out that their pump was ATP-independent. Naming of the variable *h*, taken from the model by Li and Rinzel (1994), was also presented in a contradictory manner by several authors. The very same variable *h* was given alternative explanations, some of them having completely opposite meanings, or no explanation at all. We decided to name *h* in this work as active fraction of IP_3_Rs. Authors did not always state clearly how many astrocytes were modeled. In these cases we marked it as n/a. Some authors named gliotransmitters in their work as neurotransmitters (see e.g., Stamatakis and Mantzaris, 2006). We tried our best to give as correct a view of the models as possible.

Most of the astrocyte models presented here are based on ordinary differential equations (ODEs), modeling well-stirred astrocytes (see e.g., Nadkarni and Jung, 2003; Ullah et al., 2006; Volman et al., 2007; De Pittà et al., 2009a; Goldberg et al., 2010; Dupont et al., 2011; Valenza et al., 2011; Amiri et al., 2012a; Wade et al., 2012; Hadfield et al., 2013; Tang et al., 2013; Lallouette et al., 2014; Wallach et al., 2014; Li et al., 2016a; Chan et al., 2017; Guo et al., 2017; Handy et al., 2017; Taheri et al., 2017). These models make the assumption that chemical species have the same concentrations throughout the entire volume of the astrocyte, which can also be called as average concentrations. However, it has been shown that the concentration of certain chemical species, such as Ca^2+^, can vary drastically in different parts of the cell (see e.g., Thul, 2014; Dupont et al., 2016), and several models took into account the spatiality by modeling diffusion of at least some of the chemical species in astrocytes using partial differential equations (PDEs, see e.g., Roth et al., 1995; Höfer et al., 2002; Bennett et al., 2008a; Gibson et al., 2008; Allegrini et al., 2009; Kang and Othmer, 2009; Edwards and Gibson, 2010; Wei and Shuai, 2011; Li et al., 2012; López-Caamal et al., 2014; Mesiti et al., 2015a; Yao et al., 2018). Some models took a simpler approach to spatiality by modeling ODEs combined with fluxes between different astrocytic compartments, such as cytosol and ER (see e.g., Larter and Craig, 2005; Di Garbo et al., 2007; Postnov et al., 2007; Lavrentovich and Hemkin, 2008; Di Garbo, 2009; Zeng et al., 2009; Amiri et al., 2011a; DiNuzzo et al., 2011; Farr and David, 2011; Oschmann et al., 2017; Kenny et al., 2018). In addition of modeling Ca^2+^ fluxes between ER and cytosol, Silchenko and Tass (2008) modeled free diffusion of extracellular glutamate as a flux. It seems that most of the authors implemented their ODE and PDE models using some programming language, but a few times, for example, XPPAUT (Ermentrout, 2002) was named as the simulation tool used. Because of the stochastic nature of cellular processes (see e.g., Rao et al., 2002; Raser and O'Shea, 2005; Ribrault et al., 2011) and oscillations (see e.g., Perc et al., 2008; Skupin et al., 2008), different stochastic methods have been developed for both reaction and reaction-diffusion systems. These stochastic methods can be divided into discrete and continuous-state stochastic methods. Some discrete-state reaction-diffusion simulation tools can track each molecule individually in a certain volume with Brownian dynamics combined with a Monte Carlo procedure for reaction events (see e.g., Stiles and Bartol, 2001; Kerr et al., 2008; Andrews et al., 2010). On the other hand, the discrete-state Gillespie stochastic simulation algorithm (Gillespie, 1976, 1977) and τ-leap method (Gillespie, 2001) can be used to model both reaction and reaction-diffusion systems. A few simulation tools already exist for reaction-diffusion systems using these methods (see e.g., Wils and De Schutter, 2009; Oliveira et al., 2010; Hepburn et al., 2012). In addition, continuous-state chemical Langevin equation (Gillespie, 2000) and several other stochastic differential equations (SDEs, see e.g., Shuai and Jung, 2002; Manninen et al., 2006a,b) have been developed for reactions to ease the computational burden of discrete-state stochastic methods. A few simulation tools providing hybrid approaches also exist and they combine either deterministic and stochastic methods or different stochastic methods (see e.g., Salis et al., 2006; Lecca et al., 2017). Of the above-named methods, most realistic simulations are provided by the discrete-state stochastic reaction-diffusion methods, but none of the covered astrocyte models used these stochastic methods or available simulation tools for both reactions and diffusion for the same variable. However, four models combined stochastic reactions with deterministic diffusion in the astrocytes. Skupin et al. (2010) and Komin et al. (2015) modeled with the Gillespie algorithm the detailed IP_3_R model by De Young and Keizer (1992), had PDEs for Ca^2+^ and mobile buffers, and ODEs for immobile buffers. Postnov et al. (2009) modeled diffusion of extracellular glutamate and ATP as fluxes, had an SDE for astrocytic Ca^2+^ with fluxes between ER and cytosol, and ODEs for the rest. MacDonald and Silva (2013) had a PDE for extracellular ATP, an SDE for astrocytic IP_3_, and ODEs for the rest. In addition, a few studies modeling just reactions and not diffusion used stochastic methods (SDEs or Gillespie algorithm) at least for some of the variables (see e.g., Nadkarni et al., 2008; Postnov et al., 2009; Sotero and Martínez-Cancino, 2010; Riera et al., 2011a,b; Toivari et al., 2011; Tewari and Majumdar, 2012a,b; Liu and Li, 2013a; Tang et al., 2016; Ding et al., 2018).

## 3. Results

Previous studies in experimental and computational cell biology fields have guided the development of computational models for astrocytes and their interactions with neurons. Most of the firstly developed astrocyte models were relatively simplistic but they were gradually expanded to cover astrocytic regulation of a variety of phenomena and cells in the nervous system. Next, we will present the computational models for astrocytes in section 3.1 and the computational models that include bidirectional signaling between neurons and astrocytes in section 3.2.

### 3.1. Computational astrocyte models

The early phase of model development concentrated more on single astrocytes and astrocyte-astrocyte communication. We will go through the single astrocyte models in section 3.1.1 and the astrocyte network models in section 3.1.2.

#### 3.1.1. Single astrocyte models

Half of the single astrocyte models were so-called generic, meaning that they did not describe astrocytes in any specific anatomical brain location. Others, however, were specified to model astrocytes in the cerebrum (Farr and David, 2011; Witthoft and Karniadakis, 2012), cerebral cortex (Diekman et al., 2013; Witthoft et al., 2013; Mesiti et al., 2015b; Kenny et al., 2018), cortex (De Pittà et al., 2009b; Toivari et al., 2011), hippocampus (Riera et al., 2011a,b; Chander and Chakravarthy, 2012), as well as the visual cortex (Gibson et al., 2007; Bennett et al., 2008b) and somatosensory cortex (Bennett et al., 2008b; Taheri et al., 2017). One third of the single astrocyte models took into account neurotransmitters in a simplistic way just as a stimulus, having either the neurotransmitter as a constant, step function, or something similar (see e.g., Larter and Craig, 2005; Gibson et al., 2007; Bennett et al., 2008b; De Pittà et al., 2009a; Dupont et al., 2011; Toivari et al., 2011; Witthoft and Karniadakis, 2012; Hadfield et al., 2013; Witthoft et al., 2013; Kenny et al., 2018). Only two models (Chander and Chakravarthy, 2012; Oschmann et al., 2017) actually modeled the amount of neurotransmitter with a differential equation. The stimulus to the astrocyte model by Oschmann et al. (2017) was taken from the model by Tsodyks and Markram (1997). In addition, Mesiti et al. (2015b) modeled the presynaptic neuron. We included these three models (Chander and Chakravarthy, 2012; Mesiti et al., 2015b; Oschmann et al., 2017) under single astrocyte models, because these models did not have bidirectional communication between astrocytes and neurons. The characteristics of single astrocyte models can be found in Table 2.

**Table 2:** Characteristics of single astrocyte models.

**Model**	**No**.	**Input**	**Variables**	**Ca^2+^ fluxes**	**Diffusion**	**GJ**	**Output**	**Event**
De Young and Keizer (1992) and Li and Rinzel (1994) **-TYPE MODELS**								
Bennett et al., 2008b	1	[Glu]_syn_	[Ca^2+^], [EET]_ext_, *h*, [IP_3_]	CICR, endogenous buffer, leak from ER into cyt, SERCA	D_cyt_: [IP_3_],D_ext_: [EET]_ext_	No	[EET]_ext_	Vasc.
Chander and Chakravarthy, 2012	1	Current, [Glu]_syn_	[Ca^2+^], [EET]_ext_, [GLC], *h*, [IP_3_], [LAC]	CICR, endogenous buffer, leak from ER into cyt, SERCA	No	No	[EET]_ext_	Vasc.
De Pittà et al., 2009b	1	[IP_3_]	[Ca^2+^], *h*	CICR, leak from ER into cyt, SERCA	No	No	[Ca^2+^]	Ca^2+^
Farr and David, 2011	1	ρ, [K^+^]_N_	[Ca^2+^], [Ca^2+^]_ER_, [EET], *h*, [IP_3_], [K^+^]_ext_, *n*_BK_, *V*_m_	CICR, endogenous buffer, leak from ER into cyt, SERCA	No	No	[K^+^]_ext_	Vasc.
Gibson et al., 2007	1	[Glu]_syn_	χ, [ATP]_ext_, [Ca^2+^], G, *h*, [IP_3_], PIP_2_	CICR, endogenous and exogenous buffers, leak from ER into cyt, SERCA	D_ext_: [ATP]_ext_	No	[ATP]_ext_	Vasc.
Hadfield et al., 2013	1	[Glu]_syn_	[20-HETE], [Ca^2+^], [EET], *h*, [IP_3_]	CICR, endogenous buffer, leak from ER into cyt, SERCA	No	No	[20-HETE], [EET]	Vasc.
Kenny et al., 2018	1	[Glu]_syn_, [K^+^]_N_, [NO]_N_	[Ca^2+^], [Ca^2+^]_ER_, [EET], *h*, [${\text{HCO}}_{3}^{-}$], [IP_3_], [K^+^], [K^+^]_ext_, *m*_TRPV_, *n*_BK_, [Na^+^], [NO], *R*_vol/area_	CICR, endogenous and exogenous buffers, leak from ER into cyt, SERCA, TRPV4	No	No	[K^+^]_ext_	Vasc.
Komin et al., 2015	1	Spon.	*B*_i_, *B*_i,ER_, *B*_m_, *B*_m,ER_, [Ca^2+^], [Ca^2+^]_ER_, detailed IP_3_R	CICR, immobile and mobile buffers, leak from ER into cyt, SERCA	D_cyt_: [Ca^2+^], *B*_m_,D_ER_: [Ca^2+^]_ER_, *B*_m,ER_	No	[Ca^2+^]	Ca^2+^
	1	Spon.	[Ca^2+^], [Ca^2+^]_ER_	CICR, influx, leak from ER into cyt, MCU, mitochondrial NCX, PMCA, SERCA	No	No	[Ca^2+^]	Ca^2+^
Mesiti et al., 2015b	1	*V*_in,N_ → [IP_3_]	[Ca^2+^], [IP_3_]	CICR, leak from ER into cyt, SERCA	No	No	[Ca^2+^]	Ca^2+^
Roth et al., 1995	1	[IP_3_]	[Ca^2+^], [Ca^2+^]_ER_, *h*	CICR, leak from ER into cyt, SERCA	D_cyt_: [Ca^2+^],D_ER_: [Ca^2+^]_ER_	No	[Ca^2+^]	Ca^2+^
Skupin et al., 2010	1	Spon.	*B*_i_, *B*_m_, [Ca^2+^], detailed IP_3_R	CICR, immobile and mobile exogenous buffers, leak from ER into cyt, SERCA	D_cyt_: [Ca^2+^], *B*_m_	No	[Ca^2+^]	Ca^2+^
Witthoft and Karniadakis, 2012	1	[Glu]_syn_, [K^+^]_N_	[Ca^2+^], [EET], *h*, [IP_3_], [K^+^]_ext_, *m*_TRPV_, *n*_BK_, *V*_m_	CICR, endogenous buffer, leak from ER into cyt, SERCA, TRPV4	No	No	[K^+^]_ext_	Vasc.
Witthoft et al., 2013	1	[Glu]_syn_, [K^+^]_N_	[Ca^2+^], [EET], *h*, [IP_3_], [K^+^], [K^+^]_ext_, *m*_TRPV_, *n*_BK_, [Na^+^], *V*_m_	CICR, endogenous buffer, leak from ER into cyt, SERCA, TRPV4	No	No	[K^+^]_ext_	Vasc.
Höfer et al. (2002) **-TYPE MODELS**								
Lavrentovich and Hemkin, 2008	1	Spon.	[Ca^2+^], [Ca^2+^]_ER_, [IP_3_]	CICR, efflux, influx, leak from ER into cyt, SERCA	No	No	[Ca^2+^]	Ca^2+^
Toivari et al., 2011	1	[5-HT]_syn_	[Ca^2+^], [Ca^2+^]_ER_, [IP_3_], *R*	CCE, CICR, efflux, leak from ER into cyt, leak from ext into cyt, P2XR, SERCA	No	No	[Ca^2+^]	Ca^2+^
Zeng et al., 2009	1	Spon.	[Ca^2+^], [Ca^2+^]_ER_, H-H channel kinetics, [IP_3_]	CICR, efflux, leak from ER into cyt, SERCA, 4 types of VGCCs	No	No	[Ca^2+^]	Ca^2+^
De Young and Keizer (1992), Li and Rinzel (1994), and Höfer et al. (2002) **-TYPE MODELS**								
De Pittà et al., 2009a	1	[IP_3_]	[Ca^2+^], *h*	CICR, leak from ER into cyt, SERCA	No	No	[Ca^2+^]	Ca^2+^
	1	[IP_3_]	[Ca^2+^], *h*, [IP_3_]	CICR, leak from ER into cyt, SERCA	No	No	[Ca^2+^]	Ca^2+^
	1	[Glu]_syn_	[Ca^2+^], *h*, [IP_3_]	CICR, leak from ER into cyt, SERCA	No	No	[Ca^2+^]	Ca^2+^
Diekman et al., 2013	1	GLC, O_2,out_	ΔΨ, [ADP], [ADP]_mito_, [Ca^2+^]_ER_, [Ca^2+^]_mito_, [Ca^2+^]_ps_, *h*, [H^+^]_mito_, [IP_3_], K^+^, Na^+^, [NADH]_mito_, O_2,mito_, PTP	Buffer, CICR, leak from ER into cyt, MCU, mitochondrial NCX, SERCA	No	No	[ATP]_mito_	Home.
	1	O_2,mito_, Pyr	ΔΨ, [ADP]_mito_, [Ca^2+^]_mito_, [NADH]_mito_	MCU, mitochondrial NCX	No	No	[ATP]_mito_	Home.
Ding et al., 2018	1	Spon.	[Ca^2+^], [Ca^2+^]_ER_, H-H channel kinetics, [IP_3_], detailed IP_3_R	CCE, CICR, efflux, leak from ER into cyt, SERCA, 2 types of VGCCs, contribution of DAG/PKC to VGCCs	No	No	[Ca^2+^]	Ca^2+^
Handy et al., 2017	1	[IP_3_]	[Ca^2+^], [Ca^2+^]_free_, *h*	CCE, CICR, efflux, leak from ER into cyt, leak from ext into cyt, PMCA, SERCA	No	No	[Ca^2+^]	Ca^2+^
Oschmann et al., 2017	1	Glu_syn_	[Ca^2+^], [Ca^2+^]_ER_, *h*, [IP_3_], [K^+^], [Na^+^], *V*_m_	CICR, leak from ER into cyt, NCX, SERCA	No	No	[Ca^2+^]	Ca^2+^
Riera et al., 2011a	1	Spon.	[Ca^2+^], [Ca^2+^]_free_, *h*, [IP_3_]	CCE, CICR, efflux, influx via channels, leak from ER into cyt, SERCA	No	No	[Ca^2+^]	Ca^2+^
Riera et al., 2011b	1	Spon.	[Ca^2+^], [Ca^2+^]_free_, *h*, [IP_3_]	CCE, CICR, efflux, influx via channels, leak from ER into cyt, SERCA	No	No	[Ca^2+^]	Ca^2+^
Taheri et al., 2017	1	[IP_3_]	[Ca^2+^], [Ca^2+^]_free_, *h*	CCE, CICR, efflux, leak from ER into cyt, leak from ext into cyt, PMCA, SERCA	No	No	[Ca^2+^]	Ca^2+^
**OTHER TYPES OF MODELS**								
Dupont et al., 2011	1	[Glu]_syn_	[Ca^2+^], [DAG], [DIM], *f*_PKC_, [IP_3_], *R*_inact_	CICR, efflux, influx, leak from ER into cyt, SERCA	No	No	[Ca^2+^]	Ca^2+^
Karimi et al., 2018	1	Ca^2+^	Ca^2+^, ${\text{Ca}}_{\text{ER}}^{2+}$	CICR, efflux, leak from ER into cyt, SERCA	No	No	Ca^2+^	Ca^2+^
Larter and Craig, 2005	1	[Glu]_syn_	[Ca^2+^], [Ca^2+^]_ER_, [Glu]_ext_, [IP_3_]	CICR, efflux, Glu-dependent influx, influx, leak from ER into cyt, SERCA	No	No	[Glu]_ext_	Ca^2+^
López-Caamal et al., 2014	1	[Ca^2+^]	*B*_i_, [Ca^2+^], [Ca^2+^]_ER_	CICR, immobile endogenous and exogenous buffers, efflux via pump, influx via IP_3_-dependent channel, perturbation flux, RyR, SERCA	D_cyt_: [Ca^2+^],D_ER_: [Ca^2+^]_ER_	No	[Ca^2+^]	Ca^2+^
Montaseri and Yazdanpanah, 2014	1	Syn. act.	Ca^2+^, *S*_m_=IP_3_	Efflux, influx, IP_3_-dependent influx	No	No	Ca^2+^	Ca^2+^

*This table shows the following: model, number (No.) of astrocytes modeled, input for the astrocytes, astrocytic variables described by differential equations, astrocytic Ca^2+^ fluxes related to cytosolic Ca^2+^, diffusion of astrocytic variables either in the cytosol, ER, or extracellular space, not between these spaces, gap junction signaling (GJ) between astrocytes, output of the astrocytes, and experimentally shown event that the model was finetuned to capture [Ca^2+^ dynamics (Ca^2+^), homeostasis (Home.), and vascular (Vasc.)]. Compartment is cytosol (cyt) if not otherwise stated. Amounts modeled in concentrations are given inside square brackets. In addition to astrocytes, Gibson et al. (2007), Bennett et al. (2008b), Farr and David (2011), Chander and Chakravarthy (2012), Witthoft and Karniadakis (2012), Witthoft et al. (2013), and Kenny et al. (2018) modeled smooth muscle cells. Gibson et al. (2007) and Kenny et al. (2018) modeled also an endothelial cell, and Kenny et al. (2018) included K^+^, glutamate, and nitric oxide (NO) release from neurons. However, Gibson et al. (2007) did not provide a detailed explanation of their model, but only cited previous models. Chander and Chakravarthy (2012) modeled also neuronal membrane potential and neuronal glutamate release. The stimulus for the model by Oschmann et al. (2017) came from the model by Tsodyks and Markram (1997). Mesiti et al. (2015b) modeled also the presynaptic neuron. Diekman et al. (2013) modeled also aspects of mitochondrial signaling, cell volume, and membrane potential in the astrocyte. Zeng et al. (2009) and Ding et al. (2018) modeled Hodgkin-Huxley (H-H) type channel gating kinetics (Hodgkin and Huxley, 1952). In addition, Ding et al. (2018) simulated two models, with and without detailed IP_3_R model modified from De Young and Keizer (1992). Komin et al. (2015) presented two models, a reaction-diffusion model and a reaction model. De Pittà et al. (2009a) simulated three models, of which the simplest one is the model by Li and Rinzel (1994). However, the details of all three models are given together in the table under De Young and Keizer (1992), Li and Rinzel (1994), and Höfer et al. (2002) -type models. Diekman et al. (2013), Mesiti et al. (2015b), and Karimi et al. (2018) presented two models, the others were simplified versions of the main model. However, the simpler model by Mesiti et al. (2015b) was not detailed enough based on our criteria in section 2.2*.

Most of the single astrocyte models studied Ca^2+^ oscillations, of which a few models specifically focused on modeling only spontaneous Ca^2+^ oscillations (see Table 2). All the other models had components for CICR and SERCA pump except the model by Montaseri and Yazdanpanah (2014). Furthermore, all the other models except the models by López-Caamal et al. (2014) and Montaseri and Yazdanpanah (2014) modeled leak from the ER into the cytosol. Half of the models had influx of Ca^2+^ from outside of the astrocyte or efflux of Ca^2+^ to outside of the astrocyte. About one third of the models took into account Ca^2+^ buffers and astrocytic release of signaling molecules. None of the models had gap junctions, because these were single astrocyte models. Thus, these models had similar core structure with small variations. As an example, six modeled capacitive Ca^2+^ entry (CCE) which is mediated via store-operated Ca^2+^ (SOC) channels (Riera et al., 2011a,b; Toivari et al., 2011; Handy et al., 2017; Taheri et al., 2017; Ding et al., 2018) and two modeled VGCCs (Zeng et al., 2009; Ding et al., 2018), as well as one had Ca^2+^ flux via ryanodine receptors (RyRs) (López-Caamal et al., 2014) and three had Ca^2+^ influx via TRP vanilloid-related channel 4 (TRPV4) (Witthoft and Karniadakis, 2012; Witthoft et al., 2013; Kenny et al., 2018). Only Diekman et al. (2013) and Komin et al. (2015) modeled mitochondrial Ca^2+^ unitransporters (MCUs). Two modeled mitochondrial NCX (Diekman et al., 2013; Komin et al., 2015) and one modeled NCX via plasma membrane (Oschmann et al., 2017). Skupin et al. (2010), Komin et al. (2015), and Ding et al. (2018) used detailed IP_3_R model by De Young and Keizer (1992). Six models had diffusion either in the cytosol, in the ER, or in the extracellular space (Roth et al., 1995; Gibson et al., 2007; Bennett et al., 2008b; Skupin et al., 2010; López-Caamal et al., 2014; Komin et al., 2015).

The first models developed in this category were the models by Roth et al. (1995) and Larter and Craig (2005). Roth et al. (1995) extended the model by Li and Rinzel (1994) by adding Ca^2+^ diffusion in the cytosol and ER. They also divided their cell to alternating active and passive compartments. In passive compartments, Ca^2+^ signal propagated by diffusion, whereas in active compartments there were also additional Ca^2+^ fluxes propagating the signal. Roth et al. (1995) showed with their model that different parts of the astrocyte, such as the ER membrane, produced different frequencies of Ca^2+^ oscillations if the diffusion constant had a value close to the physiological range. If the diffusion constant had unphysiologically high value, then the different parts of the astrocyte all oscillated with the same frequency.

Larter and Craig (2005) presented the only model in this category that included a differential equation for extracellular glutamate ([Glu]_ext_). Their equation for extracellular glutamate included the rate of glutamate vesicle release from the astrocyte, the input glutamate rate arriving from nearly synapses, and two terms describing the rates of glutamate uptake by neurons and astrocytes. Larter and Craig (2005) tested their model by setting the extracellular glutamate to zero during the simulations (meaning both glutamate release from the astrocyte and glutamate arriving from the nearby synapses were zero) and showed that the astrocytic Ca^2+^ was not oscillating in this case. They further tested their model when the astrocyte was not releasing glutamate, but they still had the input glutamate rate arriving from nearly synapses. In this case, the astrocytic Ca^2+^ was oscillating. They also tested how the number of glutamate in the astrocytic vesicles affected the model behavior. They showed that when increasing the number of glutamate in the astrocytic vesicles, the Ca^2+^ oscillations transformed into Ca^2+^ bursting oscillations. Thus, their message was that the presence of glutamate, but not the positive astrocytic glutamate feedback, was required for astrocytic Ca^2+^ oscillations. In addition, the positive glutamate feedback process made it possible for the astrocyte to have bursting oscillations when the input glutamate rate arriving from nearby synapses had low values.

Only six of the single astrocyte models had K^+^ concentration modeled in the perisynaptic space, perivascular space [in Table 2 called as extracellular space (ext)], or intracellular space of the astrocyte. These are marked in Table 2 as [K^+^]_N_, [K^+^]_ext_, and [K^+^], respectively. The model by Diekman et al. (2013) had the most simple K^+^ model, they modeled only astrocytic K^+^ that depended on NKA. Oschmann et al. (2017) modeled both the astrocytic and extracellular K^+^ concentrations. The astrocytic K^+^ concentration depended on three transmembrane transporters; glutamate transporter, NKA, and NCX. The extracellular K^+^ concentration was a function of astrocytic K^+^ concentration. Farr and David (2011) and Witthoft and Karniadakis (2012) modeled the K^+^ concentration both in the perisynaptic space [named as synaptic cleft and synaptic space by Farr and David (2011) and Witthoft and Karniadakis (2012)] and in the perivascular space. They both used the same simple model for the K^+^ concentration in the perisynaptic space. K^+^ was released from the neurons into the perisynaptic space and removed from the perisynaptic space via the astrocytic NKA (Farr and David, 2011). However, Witthoft and Karniadakis (2012) named the removal so that it included both astrocytic NKA and astrocytic inwardly rectifying K^+^- and voltage-gated K^+^ (KIR) channel. Farr and David (2011) and Witthoft and Karniadakis (2012) also used the same simple model for the K^+^ concentration in the perivascular space. The K^+^ concentration in the perivascular space depended on astrocytic voltage- and Ca^2+^-operated K^+^ (BK) channels and smooth muscle cell's KIR channels. They also had a K^+^-dependent decay term in the perivascular space. Witthoft et al. (2013) modeled in more detail the K^+^ concentration in the perisynaptic space (named as synaptic space by Witthoft et al., 2013), intracellular space of the astrocyte, and perivascular space. In the model by Witthoft et al. (2013), the K^+^ concentration in the perisynaptic space depended on K^+^ released from the neurons and removed via the astrocytic NKCC1 and NKA, in addition to (bidirectional) astrocytic KIR channels and a decay term. The astrocytic K^+^ concentration depended on K^+^ entering from the perisynaptic space via NKA and NKCC1, in addition to astrocytic KIR channels on the perisynaptic side as well as astrocytic BK and KIR channels on the perivascular side of the astrocyte. The equation also had a decay term. The K^+^ concentration in the perivascular space depended on astrocytic BK and KIR channels as well as arteriolar KIR channels and a decay term. Kenny et al. (2018) modeled the K^+^ concentration in the perisynaptic space (named as synaptic cleft by Kenny et al., 2018), intracellular space of the astrocyte, perivascular space, intracellular space of the smooth muscle cell, and extracellular space. In the model by Kenny et al. (2018), the K^+^ concentration in the perisynaptic space depended on K^+^ released from the neuron and removed via the astrocytic K^+^/Cl^−^ cotransporter (KCC1), NKCC1, and NKA, in addition to K^+^ diffusion between extracellular space and perisynaptic space as well as astrocytic K^+^ channels. The astrocytic K^+^ concentration depended on K^+^ entering from the perisynaptic space via KCC1, NKCC1, and NKA, in addition to K^+^ channels on the perisynaptic side and BK channels on the perivascular side of the astrocyte. The K^+^ concentration in the perivascular space depended on astrocytic BK channels and smooth muscle cell's KIR channels. In conclusion, only the model by Witthoft et al. (2013) took into account spatial K^+^ buffering.

Some of the most recent models developed in this category were the models by Komin et al. (2015), Handy et al. (2017), and Taheri et al. (2017). Komin et al. (2015) presented two models, a reaction-diffusion model and a reaction model. With both models they tested if the temperature-dependent SERCA activity was the reason for the differences in Ca^2+^ activity. They showed that their reaction-diffusion model behaved similarly to the experimental data, thus increased SERCA activity (higher temperature) led to decreased Ca^2+^ activity. On the other hand, their reaction model showed the opposite. Thus, they claimed that spatiality was needed to be taken into account to get biologically correct results. However, since the core models were different in the reaction-diffusion and reaction models, it would be interesting to see how the results would look like if the same core model was tested with and without diffusion.

Handy et al. (2017) and Taheri et al. (2017) used the same model but explored somewhat different parameter spaces. They studied the role of SOC channels as well as the PMCA and SERCA pumps in Ca^2+^ activity. They specifically tested which form the Ca^2+^ response had with different parameter values of the channel and pumps (single peak, multiple peaks, plateau, or long-lasting response). They found out that SOC channels were necessary for plateau and long-lasting responses as well as for stable oscillations with multiple peaks. Stable oscillations disappeared when the SERCA pump was partially blocked, but plateau and long-lasting responses were still present. The likelihood of having multiple peaks increased when the PMCA pump was blocked. Taheri et al. (2017) also did Ca^2+^ imaging on cortical astrocytes in mice. They applied ATP on acute brain slices and recorded the Ca^2+^ responses from different subcompartments of the astrocytes, from soma as well as from large and short processes, and categorized the results into four different types of responses named above. Their conclusion was that the variability mainly stemmed from differences in IP_3_ dynamics and Ca^2+^ fluxes via SOC channels. To take into account the experimental variability between the different subcompartments, Taheri et al. (2017) ran simulations with different parameter values of the SOC channel and the PMCA and SERCA pumps together with the input IP_3_ kinetics. Next, they chose the parameter subspaces that matched their experimental data the best. Their simulations suggested that the soma had higher PMCA and lower SERCA flux rates as well as shorter rise duration for the IP_3_ transient than the small and large processes.

#### 3.1.2. Astrocyte network models

Half of the astrocyte network models were so-called generic. Others, however, were specified to model astrocytes in the cerebrum (Iacobas et al., 2006; Ghosh et al., 2010), cortex (Goldberg et al., 2010; Wallach et al., 2014), neocortex (Li et al., 2012), visual cortex and somatosensory cortex (Bennett et al., 2008a), hippocampus (Goto et al., 2004; Ullah et al., 2006), retina (Edwards and Gibson, 2010), spinal cord (Bennett et al., 2006; Gibson et al., 2008), as well as the striatum (Höfer et al., 2002). One fourth of the astrocyte network models took into account neurotransmitters in a simplistic way just as a stimulus, having either the glutamate as a constant, step function, or something similar (see e.g., Goto et al., 2004; Ullah et al., 2006; Bennett et al., 2008a; Kang and Othmer, 2009; MacDonald and Silva, 2013). Only Wallach et al. (2014) actually modeled the amount of neurotransmitter glutamate with a differential equation. The stimulus to the astrocyte model by Wallach et al. (2014) was taken from the model by Tsodyks and Markram (1997). We included this model under astrocyte models because this model was not bidirectional between astrocytes and neurons. The characteristics of astrocyte network models can be found in Table 3.

**Table 3:** Characteristics of astrocyte network models.

**Model**	**No**.	**Input**	**Variables**	**Ca^2+^ fluxes**	**Diffusion**	**GJ**	**Output**	**Event**
De Young and Keizer (1992) and Li and Rinzel (1994) **-TYPE MODELS**								
Bennett et al., 2005	19–57	[ATP]_ext_	χ, [ATP]_ext_, [Ca^2+^], *h*, [IP_3_]	CICR, endogenous buffer, leak from ER into cyt, SERCA	D_cyt_: [IP_3_], D_ext_: [ATP]_ext_	No	[ATP]_ext_	Ca^2+^
Bennett et al., 2006	19–95	[ATP]_ext_	χ, [ATP]_ext_, [Ca^2+^], *h*, [IP_3_]	CICR, endogenous buffer, leak from ER into cyt, SERCA	D_cyt_: [IP_3_], D_ext_: [ATP]_ext_	No	[ATP]_ext_	Ca^2+^
Bennett et al., 2008a	1–7	[Glu]_syn_	[Ca^2+^], [EET]_ext_, *h*, [IP_3_]	CICR, endogenous buffer, leak from ER into cyt, SERCA	D_cyt_: [IP_3_], D_ext_: [EET]_ext_	No	[EET]_ext_	Vasc.
Gibson et al., 2008	3–n/a	[ATP]_ext_	χ, [ATP]_ext_, [Ca^2+^], *h*, [IP_3_]	CICR, endogenous buffer, leak from ER into cyt, SERCA	D_cyt_: [IP_3_], D_ext_: [ATP]_ext_	No	[ATP]_ext_	Ca^2+^
Goto et al., 2004	200	[Glu]_syn_	[Ca^2+^], [IP_3_], detailed IP_3_R	CICR, leak from ER into cyt, SERCA	No	Ca^2+^, IP_3_	[Ca^2+^]	Ca^2+^
MacDonald and Silva, 2013	1–200	[Glu]_syn_	[ATP]_ext_, [Ca^2+^], [Ca^2+^]_ER_, *h*, [IP_3_]	CICR, endogenous buffer, leak from ER into cyt, leak from ext into cyt, PMCA, SERCA	D_ext_: [ATP]_ext_	No	[ATP]_ext_	Ca^2+^
Stamatakis and Mantzaris, 2006	1–n/a	[ATP]_ext_	[ATP]_ext_, [Ca^2+^], *h*, [IP_3_]	CICR, leak from ER into cyt, SERCA	D_cyt_: [IP_3_], D_ext_: [ATP]_ext_	No	[ATP]_ext_	Ca^2+^
Stamatakis and Mantzaris, 2007	1–n/a	[ATP]_ext_	[ATP]_ext_, [Ca^2+^], *h*, [IP_3_]	CICR, leak from ER into cyt, SERCA	D_ext_: [ATP]_ext_	No	[ATP]_ext_	Ca^2+^
Höfer et al. (2002) **-TYPE MODELS**								
Bellinger, 2005	9	[IP_3_]	[ATP]_ext_, [Ca^2+^], [Ca^2+^]_ER_, [Glu]_ext_, [IP_3_], *R*	CCE, CICR, efflux via pump, Glu-dependent ER release, Glu-dependent influx, leak from ER into cyt, leak from ext into cyt, P2XR, SERCA	No	Ca^2+^, IP_3_	[ATP]_ext_, [Glu]_ext_	Ca^2+^
Höfer et al., 2002	1–361	Rate of [PLCβ]	[Ca^2+^], [Ca^2+^]_ER_, [IP_3_], *R*	CCE, CICR, efflux via pump, leak from ER into cyt, leak from ext into cyt, SERCA	D_cyt_: [Ca^2+^], [IP_3_]	Ca^2+^, IP_3_	[Ca^2+^]	Ca^2+^
Li et al., 2012	3–300	Spon.	[Ca^2+^], [Ca^2+^]_ER_, [Ca^2+^]_ext_, H-H channel kinetics, [IP_3_], [K^+^], [K^+^]_ext_, *P*, *R*_k_	CICR, efflux, efflux via pump, leak from ER into cyt, SERCA, VGCC	D_cyt_: [Ca^2+^], [IP_3_],D_ext_: [Ca^2+^]_ext_, [K^+^]_ext_,	IP_3_	[Ca^2+^]	Ca^2+^
De Young and Keizer (1992), Li and Rinzel (1994), and Höfer et al. (2002) **-TYPE MODELS**								
Edwards and Gibson, 2010	361	[ATP]_ext_	χ, [ATP]_ext_, [Ca^2+^], *h*, [IP_3_]	CICR, endogenous and exogenous buffers, leak from ER into cyt, SERCA	D_cyt_: [Ca^2+^], [IP_3_],D_ext_: [ATP]_ext_	IP_3_	[ATP]_ext_	Ca^2+^
Ghosh et al., 2010	2	[GLC]_ext_, [Gln]_ext_, [Glu]_ext_	[Ca^2+^], *h*, [IP_3_]	CCE, CICR, efflux, leak from ER into cyt, leak from ext into cyt, SERCA	No	IP_3_	[LAC]	Vasc.
Goldberg et al., 2010	1–100	[IP_3_]	[Ca^2+^], *h*, [IP_3_]	CICR, leak from ER into cyt, SERCA	No	IP_3_	[Ca^2+^]	Ca^2+^
Kazantsev, 2009	30	Spon.	[Ca^2+^], *h*, [IP_3_]	CCE, CICR, efflux, leak from ER into cyt, leak from ext into cyt, SERCA	No	IP_3_	[Ca^2+^]	Ca^2+^
Lallouette et al., 2014	1,331	[IP_3_]	[Ca^2+^], *h*, [IP_3_]	CICR, leak from ER into cyt, SERCA	No	IP_3_	[Ca^2+^]	Ca^2+^
Matrosov and Kazantsev, 2011	1–10	Spon.	[Ca^2+^], *h*, [IP_3_]	CCE, CICR, efflux, leak from ER into cyt, leak from ext into cyt, SERCA	No	IP_3_	[Ca^2+^]	Ca^2+^
Ullah et al., 2006	1–3	[Glu]_syn_	[Ca^2+^], *h*, [IP_3_]	CCE, CICR, efflux, leak from ER into cyt, leak from ext into cyt, SERCA	No	IP_3_	[Ca^2+^]	Ca^2+^
Wallach et al., 2014	130	[Glu]_syn_	[Ca^2+^], *h*, [IP_3_]	CICR, leak from ER into cyt, SERCA	No	IP_3_	[Ca^2+^]	Ca^2+^
Wei and Shuai, 2011	169	[IP_3_]	[Ca^2+^], *h*, [IP_3_]	CICR, leak from ER into cyt, SERCA	D_cyt_: [Ca^2+^], [IP_3_]	IP_3_	[Ca^2+^]	Ca^2+^
**OTHER TYPES OF MODELS**								
Iacobas et al., 2006	625	[ATP]_ext_, [UTP]_ext_	[ATP]_ext_, [Ca^2+^], [IP_3_], [UTP]_ext_	CICR, efflux via pump, P2XR, sequestration flux	D_ext_: [ATP]_ext_, [UTP]_ext_	IP_3_	[ATP]_ext_, [UTP]_ext_	Ca^2+^
Kang and Othmer, 2009	11	Glu_syn_	[ATP]_ext_, [Ca^2+^], [CaPKC], [IP_3_], [IP_3_R]	CICR, leak from ER into cyt, SERCA	D_cyt_: [Ca^2+^], [CaPKC], [IP_3_], D_ext_: [ATP]_ext_	IP_3_	[ATP]_ext_	Ca^2+^

*This table shows the following: model, number (No.) of astrocytes modeled, input for the astrocytes, astrocytic variables described by differential equations, astrocytic Ca^2+^ fluxes related to cytosolic Ca^2+^, diffusion of astrocytic variables either in the cytosol, ER, or extracellular space, not between these spaces, gap junction signaling (GJ) between astrocytes, output of the astrocytes, and experimentally shown event that the model was finetuned to capture [Ca^2+^ dynamics (Ca^2+^) and vascular (Vasc.)]. Compartment is cytosol (cyt) if not otherwise stated. Amounts modeled in concentrations are given inside square brackets. In addition to astrocytes, Bennett et al. (2008a) modeled also smooth muscle cells and Edwards and Gibson (2010) modeled also Müller cells. Edwards and Gibson (2010) simulated their system both with and without diffusion. The stimulus for the model by Wallach et al. (2014) came from the model by Tsodyks and Markram (1997)*.

All the astrocyte network models studied Ca^2+^ waves and few models specifically addressed spontaneous Ca^2+^ waves and vascular events (see Table 3). All the models except the model by Iacobas et al. (2006) had the components for all three; CICR, leak from the ER into the cytosol, and the SERCA pump. About one fourth of the models took into account Ca^2+^ buffering. About one third of the models had either influx of Ca^2+^ from outside of the astrocyte or efflux of Ca^2+^ to outside of the astrocyte, or both. About half of the models took into account astrocytic release of signaling molecules. Thus, the models had equations mostly for extracellular ATP, but one considered equations for extracellular glutamate (Bellinger, 2005). However, none of the models presented a detailed mechanistic description of how the release occurs. More than half of the models took into account diffusion, and, especially, almost half of the models studied the ATP diffusion in the extracellular space. Three quarters of the astrocyte network models had gap junctions for IP_3_ but some models had them also for Ca^2+^. Thus, these models had similar core structure with small variations. As an example, Li et al. (2012) were the only ones that modeled K^+^ concentration, both in astrocytic and extracellular spaces, and VGCCs. Goto et al. (2004) were the only ones to use the detailed IP_3_R model by De Young and Keizer (1992). Höfer et al. (2002), Bellinger (2005), Ullah et al. (2006), Kazantsev (2009), Ghosh et al. (2010), and Matrosov and Kazantsev (2011) modeled CCE.

The first model developed in this category was the model by Höfer et al. (2002). Höfer et al. (2002) showed with their two-dimensional (19 × 19) astrocyte network model that IP_3_ permeability in gap junctions was a more important factor in intercellular Ca^2+^ waves than Ca^2+^ permeability. When blocking the IP_3_ permeability, intercellular Ca^2+^ wave propagation was prevented. However, intercellular Ca^2+^ wave propagation was not prevented when the Ca^2+^ permeability was blocked. In the model by Höfer et al. (2002), the IP_3_ concentration depended on two distinct production terms via phospholipase C (PLC), one corresponding to PLC isotype β (PLCβ) and the other to PLC isotype δ (PLCδ). Höfer et al. (2002) showed that PLCδ was needed to be modeled to get the downstream cells to respond to the stimulus with a Ca^2+^ increase.

Two of the newest models developed in this category were the models by Lallouette et al. (2014) and Wallach et al. (2014). Lallouette et al. (2014) simplified the astrocyte network model by Goldberg et al. (2010) to be able to simulate the function of a three-dimensional (11 × 11 × 11) astrocyte network. With this network of more than a thousand astrocytes, Lallouette et al. (2014) studied how the variability in the topology of gap-junction coupled astrocytes affected the intercellular Ca^2+^ wave propagation. They tested five different coupling rules and found out that these different coupling rules can be used to reproduce the variation in the experimental data. They showed that dense connectivity or having long-distance gap-junction coupled astrocytes reduced the intercellular Ca^2+^ wave propagation. Wallach et al. (2014) continued the previous study by stimulating astrocyte network by Tsodyks and Markram (1997) model. In the present study, the model by Wallach et al. (2014) is listed in the category of astrocyte network models because the astrocytes did not have an effect on the neuron. Wallach et al. (2014) demonstrated through experimental and simulation studies that there was a threshold stimulation frequency when astrocytes started to respond with Ca^2+^ oscillations. However, this threshold frequency was different for different astrocytes and it increased with the number of astrocytes coupled.

### 3.2. Computational neuron-astrocyte models

In recent years, bidirectional neuron-astrocyte communication has been the focus of much research in the field of neuroscience. Most of the existing neuron-astrocyte models concentrated on astrocytic Ca^2+^ dynamics affected by glutamate in the synaptic cleft, and reciprocal neuron-astrocyte signaling. Many of the models were presented without a specific biological or disease-related question, and the focus was on combining existing models into a new construction, or adding the authors' own model components to previously published models. Next, we will go through neuron-astrocyte synapse models in section 3.2.1 and neuron-astrocyte network models in section 3.2.2.

#### 3.2.1. Neuron-astrocyte synapse models

Neuron-astrocyte synapse models include models which have only one astrocyte and one to multiple synapses. Of the models covered in our study, about half of the neuron-astrocyte synapse models were found to be so-called generic models, in other words they were developed with no specific brain area or cell in mind. Others, however, were specified to model neuron-astrocyte synapses in the cortex or neocortex (Nadkarni and Jung, 2003, 2004; Di Garbo et al., 2007; Volman et al., 2007; DiNuzzo et al., 2011; Valenza et al., 2011; Nazari et al., 2015b, 2017; Amiri et al., 2016; Li et al., 2017), hippocampus (Nadkarni and Jung, 2005, 2007; Nadkarni et al., 2008; Tewari and Majumdar, 2012a,b; Tang et al., 2013, 2016; Tewari and Parpura, 2013; Li et al., 2016b), thalamocortical networks (Amiri et al., 2011a), or brainstem (Oku et al., 2016).

The modeling approaches for neurons varied depending on the author. Half of the studied publications utilized relatively complex biophysical neuron models, namely Hodgkin and Huxley (1952) model (Nadkarni and Jung, 2003; Sotero and Martínez-Cancino, 2010; Tewari and Majumdar, 2012a,b; Tang et al., 2013, 2016; Guo et al., 2017; Li et al., 2017), Traub et al. (1991) model or its derivative Pinsky and Rinzel (1994) model (Nadkarni and Jung, 2004, 2005, 2007; Silchenko and Tass, 2008; Tewari and Parpura, 2013; Li et al., 2016a,b), or Olufsen et al. (2003) model (Di Garbo et al., 2007; Di Garbo, 2009). Simpler phenomenological models used in the studied publications were the FitzHugh-Nagumo (FitzHugh, 1961) model (Postnov et al., 2007), leaky integrate-and-fire (LIF, Gerstner and Kistler, 2002) model (Wade et al., 2011, 2012; Amiri et al., 2016; De Pittà and Brunel, 2016; Nazari et al., 2017; Liu et al., 2018), Izhikevich (2007) model (Valenza et al., 2011; Nazari et al., 2015a,b,c), Morris and Lecar (1981) model or its derivatives (Volman et al., 2007; Amiri et al., 2011b, 2013b), and Suffczynski et al. (2004) neuronal population model (Amiri et al., 2011a). The released neurotransmitter was modeled explicitly by Nadkarni and Jung (2005, 2007), Volman et al. (2007), Nadkarni et al. (2008), Silchenko and Tass (2008), Amiri et al. (2011b, 2013b), Wade et al. (2011), Tewari and Majumdar (2012a,b), Tang et al. (2013, 2016), Tewari and Parpura (2013), Nazari et al. (2015a,b,c), De Pittà and Brunel (2016), and Li et al. (2017), however not necessarily with a differential equation. Other models utilized, for example, phenomenological transfer functions between neuronal events and astrocytic IP_3_ concentration. Several models had increased astrocytic IP_3_ concentration after the neuronal membrane potential increased over some threshold (marked in Table 4 as *V*_m,N_ → [IP_3_]). The details of the neuron-astrocyte synapse models can be found in Table 4.

**Table 4:** Characteristics of neuron-astrocyte synapse models.

**Model**	**No**.	**Input**	**Variables**	**Ca^2+^ fluxes**	**Diffusion**	**GJ**	**Output**	**Event**
De Young and Keizer (1992) and Li and Rinzel (1994) **-TYPE MODELS**								
Guo et al., 2017	1	*V*_m,N_ → [IP_3_]	[Ca^2+^], *h*, [IP_3_]	CICR, leak from ER into cyt, ATP-independent ER pump	No	No	*I*_astro_	Ca^2+^
Li et al., 2016a	1	*V*_m,N_ → [IP_3_]	[Ca^2+^], [Glu]_ext_, *h*, [IP_3_]	CICR, leak from ER into cyt, SERCA	No	No	*I*_ast_ = *c*[Glu]_ext_	Ca^2+^
Li et al., 2016b	1	*V*_m,N_ → [IP_3_]	[Ca^2+^], *f*=Glu_ext_, *h*, [IP_3_]	CICR, leak from ER into cyt, SERCA	No	No	*I*_ast_ = *cf*	Ca^2+^
Li et al., 2017	1	[GABA]_syn_	[Ca^2+^], [Glu]_ext_, *h*, [IP_3_]	CICR, leak from ER into cyt, SERCA	No	No	*I*_ast_ = *c*[Glu]_ext_	Ca^2+^
Liu et al., 2018	1	[2-AG]_post_	[Ca^2+^], [Glu]_ext_, *h*, [IP_3_]	CICR, leak from ER into cyt, SERCA	No	No	[Glu]_ext_	Plast.
Nadkarni and Jung, 2003	1	*V*_m,N_ → [IP_3_]	[Ca^2+^], *h*, [IP_3_]	CICR, leak from ER into cyt, SERCA	No	No	*I*_astro_	Ca^2+^
Nadkarni and Jung, 2004	1	*V*_m,N_ → [IP_3_]	[Ca^2+^], *h*, [IP_3_]	CICR, leak from ER into cyt, SERCA	No	No	*I*_astro_	Ca^2+^
Nadkarni and Jung, 2005	1	[Glu]_syn_	[Ca^2+^], *h*, [IP_3_]	CICR, leak from ER into cyt, SERCA	No	No	*I*_astro_	Hyper.
Nadkarni and Jung, 2007	1	[Glu]_syn_	[Ca^2+^], *h*, [IP_3_]	CICR, leak from ER into cyt, SERCA	No	No	[Ca^2+^]	Plast.
Nadkarni et al., 2008	1	[Glu]_syn_	[Ca^2+^], *h*, [IP_3_]	CICR, leak from ER into cyt, SERCA	No	No	[Ca^2+^]	Inf.
Tang et al., 2013	1	NT	[Ca^2+^], *h*, [IP_3_]	CICR, leak from ER into cyt, SERCA	No	No	*I*_astro_	Inf.
Tang et al., 2016	1	NT	[Ca^2+^], *h*, [IP_3_]	CICR, leak from ER into cyt, SERCA	No	No	*I*_astro_	Inf.
Valenza et al., 2011	1	*V*_m,N_ → [IP_3_]	[Ca^2+^], *h*, [IP_3_]	CICR, leak from ER into cyt, SERCA	No	No	*I*_astro_	Ca^2+^
Volman et al., 2007	1	*y*=Glu_syn_	[Ca^2+^], *f*=Glu_ext_, *h*, [IP_3_]	CICR, leak from ER into cyt, SERCA	No	No	*f*	Inf.
Wade et al., 2011	1	*y*=Glu_syn_	[Ca^2+^], *f*=Glu_ext_, *h*, [IP_3_]	CICR, leak from ER into cyt, SERCA	No	No	Ca^2+^, *f*	Plast.
Wade et al., 2012	1	[2-AG]_post_	[Ca^2+^], [Glu]_ext_, *h*, [IP_3_]	CICR, leak from ER into cyt, SERCA	No	No	[Glu]_ext_	Plast.
Höfer et al. (2002) **-TYPE MODELS**								
Di Garbo et al., 2007	1	*V*_m,N_ → [IP_3_]	[Ca^2+^], [Ca^2+^]_ER_, [IP_3_], *R*	CCE, CICR, efflux, leak from ER into cyt, leak from ext into cyt, P2XR, SERCA	No	No	*I*_astro_	Ca^2+^
Di Garbo, 2009	1	*V*_m,N_ → [IP_3_]	[Ca^2+^], [Ca^2+^]_ER_, [IP_3_], *R*	CCE, CICR, efflux, leak from ER into cyt, leak from ext into cyt, P2XR, SERCA	No	No	*I*_astro_	Ca^2+^
DiNuzzo et al., 2011	1	[Na^+^]	[ATP], [Ca^2+^], [Ca^2+^]_ER_, [G6P], [GAP], [GLC], [Gly], [IP_3_], [LAC], [Na^+^], [NADH],[O_2_], [PCr], [PEP], [Pyr]	CICR, leak from ER into cyt, leak from ext into cyt, NCX, PMCA, SERCA	No	No	*I*_astro_	Vasc.
De Young and Keizer (1992), Li and Rinzel (1994), and Höfer et al. (2002) **-TYPE MODELS**								
De Pittà and Brunel, 2016	1	[Glu]_syn_	γ, [Ca^2+^], [Glu]_ext_, *h*, [IP_3_], *x*	CICR, leak from ER into cyt, SERCA	No	No	[Glu]_ext_	Plast.
Tewari and Majumdar, 2012a	1	[Glu]_syn_	[Ca^2+^], *E*, [Glu]_ext_, *h*, [IP_3_], *O*_1_, *O*_2_, *O*_3_, *R*_f_	CICR, leak from ER into cyt, SERCA	No	No	[Glu]_ext_	Plast.
Tewari and Majumdar, 2012b	1	[Glu]_syn_	[Ca^2+^], *E*, [Glu]_ext_, *h*, [IP_3_], *O*_1_, *O*_2_, *O*_3_, *R*_f_	CICR, leak from ER into cyt, SERCA	No	No	[Glu]_ext_	Plast.
Tewari and Parpura, 2013	1	[Glu]_syn_	[Ca^2+^], *E*, [Glu]_ext_, *h*, [IP_3_], *O*_1_, *O*_2_, *O*_3_, *R*_f_	CICR, leak from ER into cyt, SERCA	No	No	[Glu]_ext_	Synch.
**OTHER TYPES OF MODELS**								
Amiri et al., 2011a	1	Syn. act.	Ca^2+^, ${\text{Ca}}_{\text{ER}}^{2+}$, *G*_m_, *S*_m_=IP_3_	CICR, efflux, influx, IP_3_-dependent influx, leak from ER into cyt, SERCA	No	No	*G*_m_	Synch.
Amiri et al., 2011b	1	NT	Ca^2+^, ${\text{Ca}}_{\text{ER}}^{2+}$, *G*_m_, *S*_m_=IP_3_	CICR, efflux, influx, IP_3_-dependent influx, leak from ER into cyt, SERCA	No	No	*I*_ast_ = *cG*_m_	Synch.
Amiri et al., 2013b	1	NT	Ca^2+^, ${\text{Ca}}_{\text{ER}}^{2+}$, *G*_m_, *S*_m_=IP_3_	CICR, efflux, influx, IP_3_-dependent influx, leak from ER into cyt, SERCA	No	No	$I_{\text{ast}}=c\text{C}{\text{a}}^{2+}$	Inf.
Amiri et al., 2016	1	Syn. act.	Ca^2+^, ${\text{Ca}}_{\text{ER}}^{2+}$, *G*_m_, *S*_m_=IP_3_	CICR, efflux, influx, IP_3_-dependent influx, leak from ER into cyt, SERCA	No	No	*I*_ast_ = *cG*_m_exp(−*E*_2_)	Synch.
Nazari et al., 2015a	1	NT	Ca^2+^, ${\text{Ca}}_{\text{ER}}^{2+}$, *G*_m_, *S*_m_=IP_3_	CICR, efflux, influx, IP_3_-dependent influx, leak from ER into cyt, SERCA	No	No	$I_{\text{ast}}=c\text{C}{\text{a}}^{2+}$	Inf.
	1	NT	Ca^2+^, *G*_m_, *S*_m_=IP_3_	Efflux, influx, IP_3_-dependent influx	No	No	$I_{\text{ast}}=c\text{C}{\text{a}}^{2+}$	Inf.
Nazari et al., 2015b	1	NT	Ca^2+^, ${\text{Ca}}_{\text{ER}}^{2+}$, *G*_m_, *S*_m_=IP_3_	CICR, efflux, influx, IP_3_-dependent influx, leak from ER into cyt, SERCA	No	No	*I*_ast_ = *cG*_m_	Inf.
Nazari et al., 2015c	1	NT	Ca^2+^, ${\text{Ca}}_{\text{ER}}^{2+}$, *G*_m_, *S*_m_=IP_3_	CICR, efflux, influx, IP_3_-dependent influx, leak from ER into cyt, SERCA	No	No	$I_{\text{ast}}=c\text{C}{\text{a}}^{2+}$	Inf.
Nazari et al., 2017	1	Syn. act.	Ca^2+^, ${\text{Ca}}_{\text{ER}}^{2+}$, *G*_m_=Glu_ext_, *S*_m_=IP_3_	CICR, efflux, influx, IP_3_-dependent influx, leak from ER into cyt, SERCA	No	No	*I*_ast_ = *cG*_m_exp(−*kE*_2_)	Synch.
	1	Syn. act.	Ca^2+^, IP_3_	Efflux, influx, IP_3_-dependent influx	No	No	$I_{\text{ast}}=c\text{C}{\text{a}}^{2+}\exp\left(-kE_{2}\right)$	Synch.
Oku et al., 2016	1	Syn. act.	Ca^2+^, 2 variables	Not specified	No	No	Variable	Ca^2+^
Postnov et al., 2007	1	Syn. act.	Ca^2+^, ${\text{Ca}}_{\text{ER}}^{2+}$, *G*_m_, *S*_m_=IP_3_	CICR, efflux, influx, IP_3_-dependent influx, leak from ER into cyt, SERCA, *V*_m,N_-dependent influx	No	No	*I*_ast_ = *cG*_m_	Plast.
Silchenko and Tass, 2008	1	[Glu]_syn_	[Ca^2+^], ${\left[Ca^{2+}\right]}_{\text{ER}}$, [Glu]_ext_, [IP_3_], *R*_1_, *R*_2_, *R*_3_, *R*_4_	CICR, efflux, Glu-dependent influx, influx, leak from ER into cyt, SERCA	D_ext_: [Glu]_ext_	No	[Glu]_ext_	Ca^2+^
Sotero and Martínez-Cancino, 2010	1	Syn. act.	[Ca^2+^], ${\left[Ca^{2+}\right]}_{\text{ER}}$, *G*_m_=Glu_ext_, *S*_m_=IP_3_	CICR, efflux, influx, IP_3_-dependent influx (depends also on IP_3_ in neighboring astrocytic microdomains), leak from ER into cyt, SERCA	No	No	*I*_ast_ = *cG*_m_	Synch.

*This table shows the following: model, number (No.) of astrocytes modeled, input for the astrocytes, astrocytic variables described by differential equations, astrocytic Ca^2+^ fluxes related to cytosolic Ca^2+^, diffusion of astrocytic variables either in the cytosol, ER, or extracellular space, not between these spaces, gap junction signaling (GJ) between astrocytes, output of the astrocytes, and experimentally shown event that the model was finetuned to capture [Ca^2+^ dynamics (Ca^2+^), vascular (Vasc.), synchronization (Synch.), information transfer (Inf.), plasticity (Plast.), and hyperexcitability (Hyper.)]. Compartment is cytosol (cyt) if not otherwise stated. Amounts modeled in concentrations are given inside square brackets. DiNuzzo et al. (2011) modeled also smooth muscle cells. Thalamocortical neural population model was used by Amiri et al. (2011a). Cortical spiking neural population model, which is based on the LIF model, was used by Amiri et al. (2016). Sotero and Martínez-Cancino (2010) used the dynamical mean field approximation to the system of 500,000 synapses with 13 equations per synapse. They reduced the number of differential equations to 13 (13 + 2) = 195. Nazari et al. (2015a) presented two different astrocyte models, with or without ${\text{Ca}}_{\text{ER}}^{\text{2}+}$ and fluxes via ER membrane. Amiri et al. (2016) and Nazari et al. (2017) presented three different models, one was the original and the other two were reductions of the original model. However, the details of the two reduction models by Amiri et al. (2016) were given in such a way that they did not fulfill our criteria given in section 2.2. Li et al. (2017) simulated two single astrocytes models, and also combined the more complex astrocyte model with a neuronal seizure model. Di Garbo et al. (2007) and Di Garbo (2009) simulated also just the astrocyte models with ATP stimuli, in addition to the neuron-astrocyte synapse models. $I_{astro}=\text{2.11}H\left(\text{ln}\left(\Delta Ca\right)\right)\ln\left(\Delta Ca\right)$, where H is the heaviside function and ΔCa = [Ca^2+^]−196.69(nM) (Nadkarni and Jung, 2003)*.

The neuron-astrocyte synapse models were developed to explain many different biological events as can be seen in Table 4. Examples of the studied phenomena included Ca^2+^ dynamics, plasticity, hyperexcitability, information transfer, synchronization, and vascular events. All the other models except the models by Oku et al. (2016) and Guo et al. (2017) had all astrocytic components for CICR, leak from the ER into the cytosol, and the SERCA pump. About one third of the models had influx of Ca^2+^ from outside of the astrocyte and efflux of Ca^2+^ to outside of the astrocyte. The models had neither gap junction signaling because these models had only one astrocyte nor buffers. Thus, these models had similar core structure with small variations. As an example, two modeled CCE (Di Garbo et al., 2007; Di Garbo, 2009) and one modeled efflux via the PMCA pump and NCX (DiNuzzo et al., 2011) as well as diffusion (Silchenko and Tass, 2008).

The first model developed in this category was the model by Nadkarni and Jung (2003). Nadkarni and Jung (2003) studied Ca^2+^ oscillations with a model consisting of a single astrocyte and single Hodgkin-Huxley type neuron. The astrocyte model was based on the model by Li and Rinzel (1994) but they added a differential equation for IP_3_. When the membrane potential of the neuron was higher than certain threshold, IP_3_ concentration was increased in the astrocyte. The model also included a degradation of IP_3_. The membrane potential of the neuron depended on additional inward current (*I*_astro_) that depended on astrocytic Ca^2+^. Nadkarni and Jung (2003) showed that with higher rate of IP_3_ production, the astrocytic Ca^2+^ concentration was higher. In addition, with large enough rate of IP_3_ production, the neuron oscillated spontaneously without any external stimulus. Their hypothesis was that a higher expression of metabotropic glutamate receptors (mGluRs) and the resulting spontaneous oscillations caused epilepsy.

As the exact mechanisms of signaling from astrocytes to neurons are still largely unknown or controversial, most of the models had phenomenological descriptions of gliotransmitter release. About one third of the models took into account gliotransmitter release with equations for extracellular glutamate (see Table 4). Other models used phenomenological transfer functions to relay the effect of gliotransmission to the target synaptic terminal (*I*_astro_, part of *I*_ast_, *f*, and *G*_m_). Only a few of the studied models had detailed astrocytic vesicle release model, namely the models by Silchenko and Tass (2008), Tewari and Majumdar (2012a,b), and Tewari and Parpura (2013). Silchenko and Tass (2008) modeled five vesicle states which represented different stages in the transmitter vesicle cycle. The rate of prefusion complex formation depended on astrocytic Ca^2+^ concentration which was modeled by two steps. In the first step, they simplified the equation where Ca^2+^ activated Ca^2+^-binding soluble N-ethylmaleimide-sensitive factor attachment protein receptor (SNARE) proteins by assuming that the concentration of activated SNARE-proteins was considered stationary. In the second step, they simplified the equation for the fusion of vesicles leading to an irreversible exocytosis of glutamate. However, Silchenko and Tass (2008) did not provide all the details of the model which makes the reuse of the model difficult. The models by Tewari and Majumdar (2012a,b) and Tewari and Parpura (2013) assumed, based on experimental data on cultured hippocampal astrocytes, that the binding of three Ca^2+^ ions was required for gliotransmitter release. The fusion and recycling process of the synaptic-like micro-vesicle was modeled using two differential equations that both depended on the probability that the synaptic-like micro-vesicle was ready to be released. In addition to these more detailed vesicle release models, De Pittà and Brunel (2016) modeled astrocytic glutamate exocytosis in a phenomenological way with just a few equations. They assumed that a fraction of gliotransmitter resources was available for release at any time. Then, every time astrocytic Ca^2+^ increased beyond a certain threshold, the fraction of readily releasable astrocytic glutamate resources was released into the periastrocytic space.

Two of the newest models were provided by Li et al. (2016a, 2017). However, these studies contained, to the best of our understanding, fundamental errors in the biological terminology. Basically, the model by Li et al. (2016a) was the same as presented by Nadkarni and Jung (2004), but the neuronal membrane potential depended on astrocytic glutamate, as presented by Postnov et al. (2009), instead of astrocytic Ca^2+^, as presented by Nadkarni and Jung (2004). Li et al. (2017) developed a GABA-activated astrocyte model (which they, misleadingly, termed GABAergic). The model by Li et al. (2017) is similar to the model by Li et al. (2016a), but Li et al. (2017) added a more complex differential equation for IP_3_ by taking into account both the GABA released by the interneuron and glutamate released by the astrocyte, somewhat similarly to Ullah et al. (2006), Nadkarni and Jung (2005), Volman et al. (2007), and others. The differential equations for the extracellular glutamate released by the astrocyte had similar forms as the IP_3_ equations and were somewhat similar to the equation by Wade et al. (2012). Li et al. (2016a) showed how a higher equilibrium concentration of extracellular glutamate or glutamate degradation time constant predicted a higher neuronal firing frequency and existence of epileptic seizures. Li et al. (2017), on the other hand, presented using their GABA-activated astrocyte model (misleadingly termed GABAergic) that after a 0.5 s long GABA stimulation, astrocytic Ca^2+^ oscillations were long-lasting. After combining the GABA-activated astrocyte model (misleadingly termed GABAergic) and a neuronal seizure model, they concluded that in this model, the astrocyte, through stimulating pyramidal neurons and thus increasing excitatory activity, prevented the transition from seizure activity into a normal firing activity state, which GABA alone was capable of inducing by inhibiting pyramidal neuron activity.

#### 3.2.2. Neuron-astrocyte network models

Neuron-astrocyte network models include models that have several astrocytes in addition to neurons. Half of the neuron-astrocyte network models were so-called generic models. Others, however, were specified to model neuron-astrocyte interactions in the cortex (Allegrini et al., 2009; Liu and Li, 2013a; Chan et al., 2017; Tang et al., 2017; Yao et al., 2018), hippocampus (Amiri et al., 2012a, 2013a; Mesiti et al., 2015a; Li et al., 2016c), spinal cord (Yang and Yeo, 2015), or thalamocortical networks (Amiri et al., 2012b,c).

The modeling strategies for neurons varied depending on the author. Three of the studied publications utilized Hodgkin and Huxley (1952) model (Liu and Li, 2013b; Li et al., 2016c; Yao et al., 2018) and one utilized Traub et al. (1991) model's derivative Pinsky and Rinzel (1994) model (Mesiti et al., 2015a). Simpler phenomenological models used in the studied publications were the FitzHugh-Nagumo (FitzHugh, 1961) model (Postnov et al., 2009; Hayati et al., 2016), LIF (Gerstner and Kistler, 2002) model (Liu and Li, 2013a; Naeem et al., 2015), Izhikevich (2007) model (Allegrini et al., 2009; Haghiri et al., 2016, 2017; Tang et al., 2017), Morris and Lecar (1981) model or its derivatives (Amiri et al., 2012a, 2013a; Chan et al., 2017), and Suffczynski et al. (2004) neuronal population model (Amiri et al., 2012b,c). The released neurotransmitter was modeled explicitly by Amiri et al. (2012a, 2013a), Liu and Li (2013a), Yang and Yeo (2015), Li et al. (2016c), and Yao et al. (2018). Other models utilized phenomenological transfer functions between the neurotransmitter and astrocytic IP_3_ concentration. The details of the neuron-astrocyte network models can be found in Table 5.

**Table 5:** Characteristics of neuron-astrocyte network models.

**Model**	**No**.	**Input**	**Variables**	**Ca^2+^ fluxes**	**Diffusion**	**GJ**	**Output**	**Event**
De Young and Keizer (1992) and Li and Rinzel (1994) **-TYPE MODELS**								
Amiri et al., 2012a	1–5	NT	[Ca^2+^], *f*, *h*, [IP_3_]	CICR, leak from ER into cyt, SERCA	No	IP_3_	*I*_ast_ = *cf*	Synch.
Amiri et al., 2013a	1–50	NT	[Ca^2+^], *f*, *h*, [IP_3_]	CICR, leak from ER into cyt, SERCA	No	IP_3_	*I*_ast_ = *cf*	Synch.
Chan et al., 2017	10,000	*V*_m,N_ → [IP_3_]	[Ca^2+^], *f*, *h*, [IP_3_]	CICR, leak from ER into cyt, SERCA	No	IP_3_	*I*_ast_ = *cf*	Synch.
Li et al., 2016c	50	NT	[ATP]_ext_, [Ca^2+^], [Glu]_ext_, *h*, [IP_3_]	CICR, leak from ER into cyt, SERCA	D_ext_: [ATP]_ext_, [Glu]_ext_	IP_3_	*I*_ast,ATP_ = *c*[ATP]_ext_, *I*_ast,Glu_ = *c*[Glu]_ext_	Inf.
Liu et al., 2016	1–25,000	[2-AG]_post_	[Ca^2+^], [Glu]_ext_, *h*, [IP_3_]	CICR, leak from ER into cyt, SERCA	No	IP_3_	[Glu]_ext_	Plast.
Naeem et al., 2015	1–5	[2-AG]_post_	[Ca^2+^], [Glu]_ext_, *h*, [IP_3_]	CICR, leak from ER into cyt, SERCA	No	IP_3_	[Glu]_ext_	Plast.
Yang and Yeo, 2015	28	[Glu]_syn_	[ATP]_ext_, [Ca^2+^], [Glu]_ext_, *h*, [IP_3_]	CICR, leak from ER into cyt, SERCA	D_ext_: [ATP]_ext_, [Glu]_ext_	IP_3_	[ATP]_ext_, [Glu]_ext_	Inf.
Yao et al., 2018	1–6	[ATP]_ext_, [Glu]_syn_, [Glu]_ext_	χ, [ATP]_ext_, [Ca^2+^], [Ca^2+^]_ER_, G, [Glu]_ext_, *h*, [IP_3_], [K^+^], [K^+^]_ext_, [Na^+^], [Na^+^]_ext_	Buffer, CICR, leak from ER into cyt, leak from ext into cyt, PMCA, SERCA	D_ext_: [ATP]_ext_, [K^+^]_ext_, [Na^+^]_ext_	No	[ATP]_ext_, [Glu]_ext_	Hyper.
Höfer et al. (2002) **-TYPE MODELS**								
Allegrini et al., 2009	400	*V*_m,N_ → [IP_3_]	[Ca^2+^], [IP_3_], *R*_in_	CICR, efflux via pump, SERCA	D_cyt_: [Ca^2+^], [IP_3_]	Ca^2+^, IP_3_	*I*_astro_	Synch.
De Young and Keizer (1992), Li and Rinzel (1994), and Höfer et al. (2002) **-TYPE MODELS**								
Liu and Li, 2013a	n/a	[Glu]_syn_	[Ca^2+^], [IP_3_], *R*_ac_	CICR, efflux via pump, leak from ER into cyt, SERCA	No	Ca^2+^, IP_3_	*I*_astro_	Inf.
Liu and Li, 2013b	6	*V*_m,N_ → [IP_3_]	[Ca^2+^], [IP_3_], *R*_ac_	CICR, efflux via pump, leak from ER into cyt, SERCA	No	Ca^2+^, IP_3_	*I*_astro_	Inf.
Tang et al., 2017	100	*V*_m,N_ → [IP_3_]	[Ca^2+^], *h*, [IP_3_]	CICR, leak from ER into cyt, SERCA	No	IP_3_	*I*_astro_	Hyper.
**OTHER TYPES OF MODELS**								
Amiri et al., 2012b	2	Syn. act.	Ca^2+^, ${\text{Ca}}_{\text{ER}}^{2+}$, *G*_m_, *S*_m_=IP_3_	CICR, efflux, influx, IP_3_-dependent influx, leak from ER into cyt, SERCA	No	IP_3_	*G*_m_	Hyper.
Amiri et al., 2012c	2	Syn. act.	Ca^2+^, ${\text{Ca}}_{\text{ER}}^{2+}$, *G*_m_, *S*_m_=IP_3_	CICR, efflux, influx, IP_3_-dependent influx, leak from ER into cyt, SERCA	No	IP_3_	*G*_m_	Synch.
Haghiri et al., 2016	1–99	Syn. act.	Ca^2+^, ${\text{Ca}}_{\text{ER}}^{2+}$, *G*_m_, *S*_m_=IP_3_	CICR, efflux, influx, IP_3_-dependent influx, leak from ER into cyt, SERCA, *V*_m,N_-dependent influx	No	No	*I*_ast_ = *cG*_m_, *I*_syn_ = (*k*−*cG*_m_)(*z*−*z*_0_)	Synch.
	1–99	Syn. act.	Ca^2+^, ${\text{Ca}}_{\text{ER}}^{2+}$, *S*_m_=IP_3_	CICR, efflux, influx, IP_3_-dependent influx, leak from ER into cyt, SERCA, *V*_m,N_-dependent influx	No	No	*I*_ast_ = *cG*_m_	Synch.
Haghiri et al., 2017	1–500	Syn. act.	Ca^2+^, ${\text{Ca}}_{\text{ER}}^{2+}$, *G*_a_=ATP_ext_, *G*_m_=Glu_ext_, *S*_m_=IP_3_	CICR, efflux, influx, IP_3_-dependent influx, leak from ER into cyt, SERCA, *V*_m,N_-dependent influx	No	No	*I*_ast_ = *c*_1_*G*_a_+*c*_2_*G*_m_ = *I*_ast,ATP_+*I*_ast,Glu_, *I*_syn_ = (*k*−*cG*_m_)(*z*−*z*_0_)	Synch.
Hayati et al., 2016	1-n/a	Syn. act.	Ca^2+^, ${\text{Ca}}_{\text{ER}}^{2+}$, *G*_m_, *S*_m_=IP_3_	CICR, efflux, influx, IP_3_-dependent influx, leak from ER into cyt, SERCA, *V*_m,N_-dependent influx	No	No	*I*_ast_ = *cG*_m_	Synch.
	1-n/a	Syn. act.	Ca^2+^, *G*_m_, *S*_m_=IP_3_	Efflux, influx, IP_3_-dependent influx, *V*_m,N_-dependent influx	No	No	*I*_syn_ = (*k*−*cG*_m_)(*z*−*z*_0_)+*c*_1_*G*_m_	Synch.
Mesiti et al., 2015a	1–20	[Ca^2+^], *V*_m,N_ → [IP_3_]	[Ca^2+^], [Ca^2+^]_ER_, [IP_3_]	CICR, efflux, influx, IP_3_-dependent influx, leak from ER into cyt, SERCA	D_cyt_: [Ca^2+^], [IP_3_]	IP_3_	*I*_astro_	Ca^2+^
Postnov et al., 2009	1–10	Syn. act.	Ca^2+^, ${\text{Ca}}_{\text{ER}}^{2+}$, *G*_a_=ATP_ext_, *G*_m_=Glu_ext_, *S*_m_=IP_3_	CICR, efflux, influx, IP_3_-dependent influx, leak from ER into cyt, SERCA, *V*_m,N_-dependent influx	D_ext_: ATP_ext_, Glu_ext_	Ca^2+^, IP_3_	*I*_ast,ATP_ = *cG*_a_, *I*_ast,Glu_ = *cG*_m_	Ca^2+^
Soleimani et al., 2015	1–24	Syn. act.	Ca^2+^, ${\text{Ca}}_{\text{ER}}^{2+}$, *G*_m_, *S*_m_=IP_3_	CICR, efflux, influx, IP_3_-dependent influx, leak from ER into cyt, SERCA	No	No	*I*_ast_	Synch.

*This table shows the following: model, number (No.) of astrocytes modeled, input for the astrocytes, astrocytic variables described by differential equations, astrocytic Ca^2+^ fluxes related to cytosolic Ca^2+^, diffusion of astrocytic variables either in the cytosol, ER, or extracellular space, not between these spaces, gap junction signaling (GJ) between astrocytes, output of the astrocytes, and experimentally shown event that the model was finetuned to capture [Ca^2+^ dynamics (Ca^2+^), synchronization (Synch.), information transfer (Inf.), plasticity (Plast.), and hyperexcitability (Hyper.)]. Compartment is cytosol (cyt) if not otherwise stated. Amounts modeled in concentrations are given inside square brackets. Liu and Li (2013b) modeled a triple-neuron feedforward-loop neuronal network. Thalamocortical neural population model was used by Amiri et al. (2012b,c). The presentation of the model by Mesiti et al. (2015a) was confusing. They seemed to present several models but the details were not given clearly. They seemed to have variables that were not used in the equations. Thus, it was difficult to know the actual model components. They simulated their model both with and without diffusion. Amiri et al. (2013a) simulated two models, the one was similar to their earlier neuron-astrocyte synapse model (Amiri et al., 2011b), and thus the details are not given here. Soleimani et al. (2015) and Haghiri et al. (2016, 2017) presented two different models, the other ones were reductions of the main ones. However, the simplified models by Soleimani et al. (2015) and Haghiri et al. (2017) were not detailed enough based on our criteria in section 2.2. Hayati et al. (2016) presented three different models, of which two models were detailed enough. A few models did not detail the mechanisms by which astrocytes communicated with each other (Haghiri et al., 2016, 2017; Hayati et al., 2016; Soleimani et al., 2015), thus it is possible that in some of these models each astrocyte is only connected to neurons (see e.g., Haghiri et al., 2017; Soleimani et al., 2015). $I_{astro}=\text{2.11}H\left(\text{ln}\left(\Delta Ca\right)\right)ln\left(\Delta Ca\right)$, where H is the heaviside function and ΔCa = [Ca^2+^]−196.69(nM) (Nadkarni and Jung, 2003)*.

The neuron-astrocyte network models were developed to explain many different biological events as can be seen in Table 5. Examples included Ca^2+^ dynamics, synchronization, information transfer, plasticity, and hyperexcitability. All the other models except the model by Allegrini et al. (2009) had components for all three; CICR, leak from the ER into the cytosol, and the SERCA pump. More than half of the models had influx of Ca^2+^ from outside of the astrocyte and efflux of Ca^2+^ to outside of the astrocyte. About one third of the models took into account gliotransmitter release by modeling extracellular glutamate, and few were also modeling extracellular ATP. Other models used phenomenological transfer functions to relay the effect of gliotransmission to the target synaptic terminal (*I*_astro_, *I*_syn_, part of *I*_ast_, and *G*_m_). None of the studied models had a detailed astrocytic vesicle release model. Most of the models had gap junction signaling for IP_3_, and some also for Ca^2+^. Thus, these models had a similar core structure with small variations. As an example, only Yao et al. (2018) modeled buffering as well as astrocytic and extracellular K^+^. Diffusion was taken into account in the models by Allegrini et al. (2009), Postnov et al. (2009), Mesiti et al. (2015a), Yang and Yeo (2015), Li et al. (2016c), and Yao et al. (2018). Yao et al. (2018) presented one of the available models for cortical spreading depression.

One of the first models developed in this category was the two-dimensional model by Postnov et al. (2009). They studied how different lengths of stimulus affected astrocytic Ca^2+^ and showed how short stimulus of less than 100 s did not induce Ca^2+^ wave propagation. However, a longer stimulus of 320 s showed Ca^2+^ wave propagation for a short distance and a stimulus of about 2,000 s showed Ca^2+^ wave propagation along the astrocyte network. They also tested how Ca^2+^ wave propagation was affected by different noise levels added to the model. They found out that the stronger the noise, the more accelerated was the Ca^2+^ wave propagation. With the largest noise level they tested, they found out that the spatially synchronized behavior was destroyed, and the model started to behave irregularly.

A few publications presented simplification of model complexity. Simplification is, in general, used to reduce the model order to allow cost-effective computation yet preserving the major, key dynamical behavior of the model. Soleimani et al. (2015), Haghiri et al. (2016, 2017), and Hayati et al. (2016) presented the original and simplified versions of the earlier published models by Postnov et al. (2007, 2009). However, most of the reduced astrocyte models were not detailed enough based on our criteria in section 2.2. In the future, it is important to put more emphasis on the model order reduction of the complex neuron-astrocyte interaction models to be able to simulate the behavior of large networks biologically more accurately (see e.g., Lehtimäki et al., 2017).

One of the newest models was the model by Chan et al. (2017). Their neuron-astrocyte network model was able to generate ultra-slow neuronal oscillatory activity patterns with frequencies less than 0.01 Hz, recorded by high-density microelectrode arrays and examined using methods reflecting conventional EEG analysis rather than conventional microelectrode array analysis. Their model showed that the frequency of these neuronal oscillations depended on the astrocytic Ca^2+^ oscillations. These astrocytic Ca^2+^ oscillations depended on the properties of IP_3_Rs and lasted from a few seconds to minutes. The results of Chan et al. (2017) also suggested that astrocytes preferred asynchronous neuronal firing to synchronous neuronal firing.

### 3.3. Model evolution

**Figure 1:**
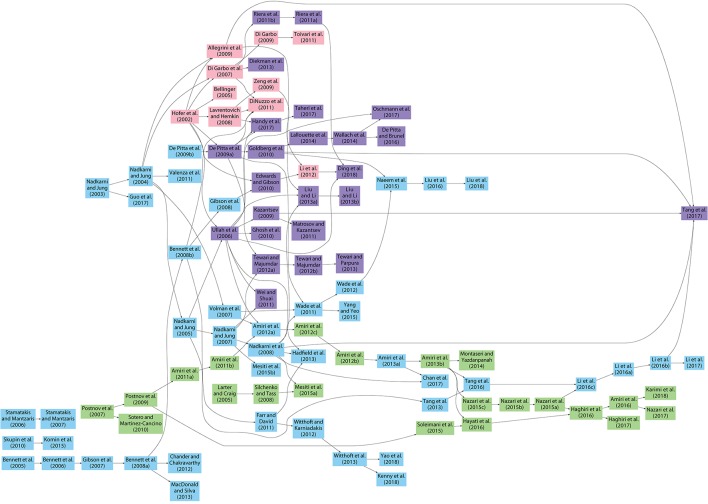
Evolution of astrocyte and neuron-astrocyte models published from 1995 to 2017. The starting point of an arrow represents the model which was used as a reference by the latter model indicated as the arrowhead. The number of arrows was minimized so that the minimum number of arrows are pointing to the arrowheads. This means basically that all the previous models in the same chain of arrows might have been used to built the model in the arrowhead, but of course not all of them probably were necessary. With blue, we presented De Young and Keizer (1992) and Li and Rinzel (1994) -type models. With pink, we presented Höfer et al. (2002) -type models. With purple, we presented De Young and Keizer (1992), Li and Rinzel (1994), and Höfer et al. (2002) -type models. All the other types of models appear green.

Figure 1 shows how the computational astrocyte and neuron-astrocyte models have evolved from each other (see also, Manninen et al., 2018). We realized that several similar or exactly the same models have been published without clearly explaining the similarity and without including citations to the other, earlier published similar models. This is both negligent and unethical. The very rules of science should be that every publication includes a description of what has been done before and what has been done now on top of the previously published publications. We have tried our best to give here as complete a picture as possible. The starting point of an arrow represents the model which was used as a reference by the latter model indicated as the arrowhead. We minimized the number of arrows so that the minimum number of arrows are pointing to the arrowheads. This means basically that all the previous models in the same chain of arrows might have been used to built the model in the arrowheads, but of course not all of them probably were necessary. Models are excluded from Figure 1 if there was no clear evidence that the authors had used any other models presented in this study as a reference and their model was not used as a reference by any other models presented in this study. The models by De Young and Keizer (1992), Li and Rinzel (1994), and Höfer et al. (2002) were most often used as a starting point when developing new models (Manninen et al., 2018). In addition, the other models that have helped to steer the field are the models by Nadkarni and Jung (2003), Bennett et al. (2005), Volman et al. (2007), De Pittà et al. (2009a), Postnov et al. (2009), and Lallouette et al. (2014).

### 4. Discussion and conclusions

In this review, we present the state-of-the-art in the computational modeling of astrocytes and the interactions astrocytes have with vascular and neuronal systems. We were delighted to see the number of researchers presently interested in modeling astrocytic functions. Due to the large number of models, we concentrated on about a hundred models that included biophysical descriptions for Ca^2+^ signaling and dynamics in astrocytes. The models were categorized into four groups: single astrocyte models, astrocyte network models, neuron-astrocyte synapse models, and neuron-astrocyte network models. We characterized the components of the models in detail, contrasted and compared the models with one another, and evaluated their usability in future work. Selecting the best models to be utilized in one's own research can be very tedious and time-consuming due to the large number of available models. We therefore wish to provide practical help and guidelines in choosing the proper astrocyte models for future modeling projects. Our work also promotes the transparency of scientific work and recommends actions to establish good practices in computational modeling and the presentation of results.

One of the fundamental questions in neuroscience is how different mechanisms of astrocytes and neuron-astrocyte interactions are linked with cognitive functions and behavior in mammals. A variety of evidence is accumulating on the roles of astrocytes in neuronal excitability, synaptic transmission, plasticity, and in higher cognitive functions, including the initiation, maintenance and consolidation of memories (Volterra et al., 2014; Magistretti and Allaman, 2015; Bazargani and Attwell, 2016). Most of the evidence stems from *in vitro* experimental studies, but also from *in vivo* studies. Experimental wet-lab work on astrocytes has given rise to a variety of studies to computationally address astrocytes' Ca^2+^ excitability and its putative role in neural functions in a variety of conditions (Manninen et al., 2018). Although there is partial controversy and ongoing debate on the existence of gliotransmission *in vitro* and *in vivo* (see Fiacco and McCarthy, 2018; Savtchouk and Volterra, 2018, and section 2.1.4), recent attempts to model the astrocytes' roles in synaptic and network dynamics are a welcome and useful additional tool to help testing various hypotheses. This is indeed a good sign: astrocytes, the important but mostly neglected glial cells, are gradually being taken into account in efforts to understand the roles of astrocytes in neural network dynamics. Based on our evaluation, we found out that *in silico* models have been presented for many of the above-mentioned, experimentally observed neural phenomena. However, it was not always clear which of the models were based on cell culture and which ones on slice studies. Very few of the models were constructed using *in vivo* data. According to our evaluation of 106 models, 53 models were constructed to study Ca^2+^ dynamics and 15 models were constructed to study neural synchronization. In addition, 12 models were used to study information transfer, 11 models were used to study vascular events, 10 models were used to study plasticity, four models were used to study hyperexcitability, and one model was used to study homeostasis. However, the models often described a limited set of molecular mechanisms which sometimes led us to doubt if the models were detailed enough to answer the questions asked.

Even though some promising studies on the modeling of glial functions exist (see e.g., Höfer et al., 2002; Nadkarni et al., 2008; De Pittà et al., 2009a; Lallouette et al., 2014; Taheri et al., 2017), the actual value of the models can only be assessed with time and the reimplementation and resimulation of published models. Astrocytic mechanisms, such as astrocytic Ca^2+^ fluxes related to cytosolic Ca^2+^, diffusion of astrocytic variables either in the cytosol, ER, or extracellular space, and gap junction signaling between astrocytes, were characterized in more detail here than in our other, educational study (Manninen et al., 2018) to facilitate the viewing of the similarities and differences of the models, as well as to support the utilization of the models in future work. In the present study, a careful review of the details of the published models revealed that several publications gave inaccurate descriptions of the models. Examples include misleading or completely missing graphical illustrations of the models, incorrect mathematical equations, biologically incorrectly or sometimes misleadingly named variables, unclear or non-existent statements of the number of cells modeled, and non-existent description of the applicability of the selected model components (see also, Manninen et al., 2018). Moreover, our detailed evaluation revealed that most models were generated making slight variations to a small set of older models that did not originally represent data obtained for astrocytes. However, neither citations to previous models with similar core structure nor explanations about what exactly was added to the previous models were provided. This made it possible, in some cases, to publish the same or a very similar model several times. Very few models provided a detailed sensitivity analysis, that is, an analysis of the robustness of the model against changes in parameter values. We therefore conclude that most of the models published thus far do not serve the scientific community in their best potential and the simulation results of the models are very difficult to reproduce. A proper validation of the simulation results against experimental findings and a careful review process of manuscripts are needed to promote the transparency and utility of *in silico* models. Large-scale neuroscience projects, such as presented by Markram et al. (2015), Amunts et al. (2016), and Grillner et al. (2016), are seeking to solve these challenges by providing sophisticated informatics tools for the construction, estimation and validation of models.

Our study highlights the need for reproducible research, which is an enormous challenge in all areas of science (Baker, 2016; Munafò et al., 2017; Rougier et al., 2017). In our other studies, we have shown how tedious and difficult it is to reproduce and replicate the simulation results of published astrocyte models (Manninen et al., 2017, 2018). We have shown that it is often impossible to reproduce the results without first carefully assessing and verifying all equations or contacting the authors for more details of the published model. In our previous studies, we have reimplemented altogether seven astrocyte models and were able to reproduce the simulation results of only two of the publications completely, based on the information in the original publications and corrigenda (Manninen et al., 2017, 2018). After fixing the observed errors in the original equations, we were able to reproduce the original results of one more model completely (Manninen et al., 2017). One of the goals of the present study is to show how many similar models have already been developed and how emphasis should be put on making the developed models usable for other researchers by publishing the model codes online. Furthermore, reviewers should be able to verify that the implementation and equations presented in the manuscript match. One solution would be to submit all the details of the model, such as equations, parameter values, initial values, and stimuli, in table format with the manuscript, similarly to what was presented in our previous studies (see e.g., Manninen et al., 2017). It would also be helpful to present the outline of the model in a table (see e.g., Tables 2–5 and Manninen et al., 2010, 2018). Reproducible models can truly advance the computational fields of neuroscience and glioscience.

It seems increasingly evident that astrocytes modulate a variety of events in the brain. Astrocytes are fully integrated into the brain cellular circuitry and are an essential part of the neural networks. Future mathematical and computational models of brain functions should better take into account the hidden capacity of astrocytes and other glial cells, as also pointed out by Sompolinsky (2014), and available methodologies (see e.g., Stiles and Bartol, 2001; Kerr et al., 2008; Skupin et al., 2008; Wils and De Schutter, 2009; Andrews et al., 2010; Oliveira et al., 2010; Hepburn et al., 2012; Tilūnaitė et al., 2017). Such comprehensive models will help us detail the complex interactions between different types of glial cells and other systems in the brain, including the vascular and neuronal systems, and may lead to a better understanding of the roles astrocytes have in the brain. Most urgently, *in silico* modeling in neuroscience should strive to incorporate state-of-the-art wet-lab data on glial cells to advance the construction and validation of models. In this process, more emphasis should be put on the selection of the animal species, the developmental stage of an organism, and the type of wet-lab preparation. Future work should also include the development of theory, based on constraints of physics and chemistry, to provide a linkage between the phenomena measured at different levels and scales in the brain. In conclusion, the description of the state-of-the-art in neuron-glia modeling and the constructive criticism presented in this work will be useful in setting new goals and guidelines for future modeling in neuroscience.

## Author contributions

TM designed the study, acquired the *in silico* models, characterized and evaluated the models in detail, tabulated the details of the models, drafted the first version of the manuscript, and coordinated the production of the final version. RH contributed to the design of the study, interpreted biological terminology used in *in silico* models, evaluated some of the models, as well as contributed to the drafting and critical revision of the manuscript. M-LL conceived and designed the study, acquired funding, interpreted concepts in *in silico* models, contributed to the drafting and critical revision of the manuscript, and supervised the study. All approved the final version of the manuscript.

### Conflict of interest statement

The authors declare that the research was conducted in the absence of any commercial or financial relationships that could be construed as a potential conflict of interest.

## Abbreviations

2-AG: 2-arachidonoylglycerol
5-HT: 5-hydroxytryptamine
20-HETE: 20-hydroxyeicosatetraenoic acid
ΔΨ: Electrochemical gradient
γ: Fraction of ligand-bound mGluRs
ρ: Ratio of bound to total Glu receptors
χ: Depletion of ATP stores
ADP: Adenosine diphosphate
ATP: Adenosine triphosphate
*B*_i_: Immobile buffer concentration
*B*_m_: Mobile buffer concentration
BK: Voltage and Ca^2+^-operated K^+^ channel
*c*: Constant
*c*_1_: Constant
*c*_2_: Constant
Ca^2+^: Calcium ion
[Ca^2+^]_free_: Total free Ca^2+^ concentration
CaPKC: Ca^2+^-PKC complex
CCE: Capacitive Ca^2+^ entry
CICR: Ca^2+^-induced Ca^2+^ release
Cl^−^: Chloride ion
cyt: Cytosol
D_cyt_: Diffusion in cyt
D_ER_: Diffusion in ER
D_ext_: Diffusion in ext
DAG: Diacylglycerol
DIM: Ligand-bound mGluR dimer
*E*: Fraction of effective synaptic-like micro-vesicles in extrasynaptic space
*E*_2_: Euclidean distance between the stimulating electrode and individual neuron
EAAT: Excitatory amino acid transporter
EET: Epoxyeicosatrienoic acid
ER: Endoplasmic reticulum
ext: Extracellular space (can be also periastrocytic, perisynaptic, extrasynaptic, or perivascular space)
*f*: Phenomenological variable modeling events from Ca^2+^ rise to vesicle release
*f*_PKC_: Fraction of active PKC
G: G protein
*G*_a_: Astrocytic mediator
*G*_m_: Astrocytic mediator
G6P: GLC 6-phosphate
GABA: Gamma-aminobutyric acid
GAP: Glyceraldehyde 3-phosphate
GJ: Gap junction
GLC: Glucose
Gln: Glutamine
Glu: Glutamate
Gly: Glycogen
*h*: Active fraction of IP_3_Rs (Li and Rinzel, 1994)
H^+^: Hydrogen ion
${\text{HCO}}_{3}^{-}$: Bicarbonate ion
H-H: Hodgkin-Huxley
Home.: Homeostasis
Hyper.: Hyperexcitability
*I*_ast_: Modulating current from the astrocyte to the neuron
*I*_ast,ATP_: Modulating current from the astrocyte to the neuron
*I*_ast,Glu_: Modulating current from the astrocyte to the neuron
*I*_astro_: Modulating current from the astrocyte to the neuron depending on astrocytic Ca^2+^ (Nadkarni and Jung, 2003)
*I*_syn_: Synaptic current
Inf.: Information transfer
IP_3_: Inositol trisphosphate
IP_3_R: IP_3_ receptor
*k*: Constant
K^+^: Potassium ion
KCC1: K^+^/Cl^−^ cotransporter
KIR: Inwardly rectifying K^+^- and voltage-gated K^+^ channel
LAC: Lactate
LIF: Leaky integrate-and-fire
mito: Mitochondrial component
*m*_TRPV_: Open probability of TRPV4 channel
MCU: Mitochondrial Ca^2+^ unitransporter
mGluR: Metabotropic Glu receptor
N: Neuron
*n*_BK_: Open BK channel probability
Na^+^: Sodium ion
NADH: Nicotinamide adenine dinucleotide hydrogen
NCX: Na^+^/Ca^2+^ exchanger
NKA: Na^+^/K^+^-ATPase
NKCC1: Na^+^/K^+^/Cl^−^ cotransporter
NMDAR: N-methyl-D-aspartate receptor
NO: Nitric oxide
No.: Number
NT: Neurotransmitter
*O*_1_–*O*_3_: Opening probabilities of three gates related to gliotransmitter release
O_2_: Oxygen
O_2,out_: O_2_ level outside the mitochondrion
ODE: Ordinary differential equation
*P*: Recovery for K^+^ in cyt
P2XR: Ionotropic purinergic ATP receptor
PCr: Phosphocreatine
PDE: Partial differential equation
PEP: Phosphoenolpyruvate
PIP_2_: Phosphatidylinositol 4,5-bisphosphate
PKC: Protein kinace C
Plast.: Plasticity
PLC: Phospholipase C
PLCβ: PLC isotype β
PLCδ: PLC isotype δ
PMCA: Plasma membrane Ca^2+^-ATPase
post: Postsynaptic
ps: Pore-space
PTP: Permeability transition pore
Pyr: Pyruvate
*R*: Active fraction of IP_3_Rs (Höfer et al., 2002)
*R*_1_–*R*_5_: Concentrations of five vesicle states
*R*_ac_: Fraction of IP_3_Rs that are not inactivated by Ca^2+^ [marked as *h* by Liu and Li (2013a) and *q* by Liu and Li (2013b), and same equation as by Allegrini et al. (2009), modified from Li and Rinzel (1994) and Höfer et al. (2002)]
*R*_f_: Fraction of the readily releasable synaptic-like micro-vesicles inside the astrocyte
*R*_in_: Fraction of IP_3_Rs that are not activated by Ca^2+^ [marked as *h* by (Allegrini et al., 2009) and same equation as by Liu and Li (2013a, b), modified from Li and Rinzel (1994) and Höfer et al. (2002)]
*R*_inact_: Fraction of Ca^2+^-inhibited IP_3_Rs (Dupont et al., 2011)
*R*_k_: Recovery for [K^+^]_ext_ (variable that restores [K^+^]_ext_ to the normal level)
*R*_vol/area_: Volume-area ratio
RyR: Ryanodine receptor
*S*_m_: Secondary messenger
SDE: Stochastic differential equation
SERCA: Sarco/ER Ca^2+^-ATPase
SIC: NMDAR-dependent slow inward current
SNARE: Soluble N-ethylmaleimide-sensitive factor attachment protein receptor
SOC: Store-operated Ca^2+^ channel
Spon.: Spontaneous
syn: Synaptic cleft
Syn. act.: Synaptic activity
Synch.: Synchronization
TRP: Transient receptor potential channel
TRPV4: TRP vanilloid-related channel 4
UTP: Uridine-5′-triphosphate
*V*_in_: Pulse membrane voltage
*V*_m_: Membrane voltage
Vasc.: Vascular
VGCC: Voltage-gated Ca^2+^ channel
*x*: Fraction of gliotransmitter resources
*y*: Fraction of synaptic resources in the active states
*z*: Synaptic activation variable
*z*_0_: Reference level of *z*.

## References

[B1] Aguado F. Espinosa-Parrilla J. F. Carmona M. A. Soriano E. (2002). Neuronal activity regulates correlated network properties of spontaneous calcium transients in astrocytes *in situ*. J. Neurosci. 22, 9430–9444. 12417668 10.1523/JNEUROSCI.22-21-09430.2002 PMC6758057

[B2] Agulhon C. Fiacco T. A. McCarthy K. D. (2010). Hippocampal short- and long-term plasticity are not modulated by astrocyte Ca^2+^ signaling. Science 327, 1250–1254. 10.1126/science.1184821 20203048

[B3] Agulhon C. Petravicz J. McMullen A. B. Sweger E. J. Minton S. K. Taves S. R.. (2008). What is the role of astrocyte calcium in neurophysiology? Neuron 59, 932–946. 10.1016/j.neuron.2008.09.004 18817732 PMC3623689

[B4] Allegrini P. Fronzoni L. Pirino D. (2009). The influence of the astrocyte field on neuronal dynamics and synchronization. J. Biol. Phys. 35, 413–423. 10.1007/s10867-009-9166-8 19669414 PMC2750747

[B5] Amiri M. Amiri M. Nazari S. Faez K. (2016). A new bio-inspired stimulator to suppress hyper-synchronized neural firing in a cortical network. J. Theor. Biol. 410, 107–118. 10.1016/j.jtbi.2016.09.007 27620666

[B6] Amiri M. Bahrami F. Janahmadi M. (2011a). Functional modeling of astrocytes in epilepsy: a feedback system perspective. Neural Comput. Appl. 20, 1131–1139. 10.1007/s00521-010-0479-0

[B7] Amiri M. Bahrami F. Janahmadi M. (2012a). Functional contributions of astrocytes in synchronization of a neuronal network model. J. Theor. Biol. 292, 60–70. 10.1016/j.jtbi.2011.09.013 21978738

[B8] Amiri M. Bahrami F. Janahmadi M. (2012b). Modified thalamocortical model: a step towards more understanding of the functional contribution of astrocytes to epilepsy. J. Comput. Neurosci. 33, 285–299. 10.1007/s10827-012-0386-8 22382677

[B9] Amiri M. Bahrami F. Janahmadi M. (2012c). On the role of astrocytes in epilepsy: a functional modeling approach. Neurosci. Res. 72, 172–180. 10.1016/j.neures.2011.11.006 22138615

[B10] Amiri M. Hosseinmardi N. Bahrami F. Janahmadi M. (2013a). Astrocyte-neuron interaction as a mechanism responsible for generation of neural synchrony: a study based on modeling and experiments. J. Comput. Neurosci. 34, 489–504. 10.1007/s10827-012-0432-6 23661228

[B11] Amiri M. Montaseri G. Bahrami F. (2011b). On the role of astrocytes in synchronization of two coupled neurons: a mathematical perspective. Biol. Cybern. 105, 153–166. 10.1007/s00422-011-0455-5 21935706

[B12] Amiri M. Montaseri G. Bahrami F. (2013b). A phase plane analysis of neuron-astrocyte interactions. Neural Netw. 44, 157–165. 10.1016/j.neunet.2013.03.018 23685459

[B13] Amunts K. Ebell C. Muller J. Telefont M. Knoll A. Lippert T. (2016). The human brain project: creating a European research infrastructure to decode the human brain. Neuron 92, 574–581. 10.1016/j.neuron.2016.10.046 27809997

[B14] Andrews S. S. Addy N. J. Brent R. Arkin A. P. (2010). Detailed simulations of cell biology with Smoldyn 2.1. PLoS Comput. Biol. 6:e1000705. 10.1371/journal.pcbi.1000705 20300644 PMC2837389

[B15] Araque A. Carmignoto G. Haydon P. G. Oliet S. H. R. Robitaille R. Volterra A. (2014). Gliotransmitters travel in time and space. Neuron 81, 728–739. 10.1016/j.neuron.2014.02.007 24559669 PMC4107238

[B16] Araque A. Parpura V. Sanzgiri R. P. Haydon P. G. (1999). Tripartite synapses: glia, the unacknowledged partner. Trends Neurosci. 22, 208–215. 10.1016/S0166-2236(98)01349-6 10322493

[B17] Backus K. H. Kettenmann H. Schachner M. (1989). Pharmacological characterization of the glutamate receptor in cultured astrocytes. J. Neurosci. Res. 22, 274–282. 10.1002/jnr.490220307 2540340

[B18] Baker M. (2016). 1,500 scientists lift the lid on reproducibility. Nature 533, 452–454. 10.1038/533452a 27225100

[B19] Bao X. Lee S. C. Reuss L. Altenberg G. A. (2007). Change in permeant size selectivity by phosphorylation of connexin 43 gap-junctional hemichannels by PKC. Proc. Natl. Acad. Sci. U.S.A. 104, 4919–4924. 10.1073/pnas.0603154104 17360407 PMC1817834

[B20] Bazargani N. Attwell D. (2016). Astrocyte calcium signaling: the third wave. Nat. Neurosci. 19, 182–189. 10.1038/nn.4201 26814587

[B21] Bellinger S. (2005). Modeling calcium wave oscillations in astrocytes. Neurocomputing 65-66, 843–850. 10.1016/j.neucom.2004.10.081

[B22] Beltrán-Castillo S. Olivares M. J. Contreras R. A. Zúñiga G. Llona I. von Bernhardi R.. (2017). D-serine released by astrocytes in brainstem regulates breathing response to CO_2_ levels. Nat. Commun. 8, 838. 10.1038/s41467-017-00960-3 29018191 PMC5635109

[B23] Bennett M. R. Buljan V. Farnell L. Gibson W. G. (2006). Purinergic junctional transmission and propagation of calcium waves in spinal cord astrocyte networks. Biophys. J. 91, 3560–3571. 10.1529/biophysj.106.082073 16905605 PMC1614486

[B24] Bennett M. R. Farnell L. Gibson W. G. (2005). A quantitative model of purinergic junctional transmission of calcium waves in astrocyte networks. Biophys. J. 89, 2235–2250. 10.1529/biophysj.105.062968 16055527 PMC1366726

[B25] Bennett M. R. Farnell L. Gibson W. G. (2008a). Origins of blood volume change due to glutamatergic synaptic activity at astrocytes abutting on arteriolar smooth muscle cells. J. Theor. Biol. 250, 172–185. 10.1016/j.jtbi.2007.08.024 17920632

[B26] Bennett M. R. Farnell L. Gibson W. G. (2008b). Origins of the BOLD changes due to synaptic activity at astrocytes abutting arteriolar smooth muscle. J. Theor. Biol. 252, 123–130. 10.1016/j.jtbi.2008.01.022 18339404

[B27] Bezprozvanny I. Watras J. Ehrlich B. E. (1991). Bell-shaped calcium-response curves of Ins(1,4,5)P_3_- and calcium-gated channels from endoplasmic reticulum of cerebellum. Nature, 351, 751–754. 10.1038/351751a0 1648178

[B28] Bezzi P. Volterra A. (2001). A neuron–glia signalling network in the active brain. Curr. Opin. Neurobiol. 11, 387–394. 10.1016/S0959-4388(00)00223-3 11399439

[B29] Bushong E. A. Martone M. E. Jones Y. Z. Ellisman M. H. (2002). Protoplasmic astrocytes in CA1 stratum radiatum occupy separate anatomical domains. J. Neurosci. 22, 183–192. 11756501 10.1523/JNEUROSCI.22-01-00183.2002 PMC6757596

[B30] Cali C. Kare K. Boges D. J. Lehvaslaiho H. Magistretti P. J. (2016). Detailed morphometric analysis of a glial process in the adult rat hippocampus, in Program No. 509.22. 2016 Neuroscience Meeting Planner (San Diego, CA: Society for Neuroscience).

[B31] Cannon R. C. Gewaltig M.-O. Gleeson P. Bhalla U. S. Cornelis H. Hines M. L.. (2007). Interoperability of neuroscience modeling software: current status and future directions. Neuroinformatics 5, 127–138. 10.1007/s12021-007-0004-5 17873374 PMC2658651

[B32] Carmignoto G. Gómez-Gonzalo M. (2010). The contribution of astrocyte signalling to neurovascular coupling. Brain Res. Rev. 63, 138–148. 10.1016/j.brainresrev.2009.11.007 19948187

[B33] Chan S.-C. Mok S.-Y. Ng D. W.-K. Goh S.-Y. (2017). The role of neuron–glia interactions in the emergence of ultra-slow oscillations. Biol. Cybern. 111, 459–472. 10.1007/s00422-017-0740-z 29128889

[B34] Chander B. S. Chakravarthy V. S. (2012). A computational model of neuro-glio-vascular loop interactions. PLoS ONE 7:e48802. 10.1371/journal.pone.0048802 23185276 PMC3502400

[B35] Charles A. C. Merrill J. E. Dirksen E. R. Sandersont M. J. (1991). Intercellular signaling in glial cells: calcium waves and oscillations in response to mechanical stimulation and glutamate. Neuron 6, 983–992. 10.1016/0896-6273(91)90238-U 1675864

[B36] Cornell-Bell A. H. Finkbeiner S. M. Cooper M. S. Smith S. J. (1990). Glutamate induces calcium waves in cultured astrocytes: long-range glial signaling. Science 247, 470–473. 10.1126/science.1967852 1967852

[B37] Crook S. M. Davison A. P. Plesser H. E. (2013). Learning from the past: approaches for reproducibility in computational neuroscience, in 20 Years of Computational Neuroscience, ed Bower J. M. (New York, NY: Springer), 73–102.

[B38] Dani J. W. Chernjavsky A. Smith S. J. (1992). Neuronal activity triggers calcium waves in hippocampal astrocyte networks. Neuron 8, 429–440. 10.1016/0896-6273(92)90271-E 1347996

[B39] De Pittà M. Brunel N. (2016). Modulation of synaptic plasticity by glutamatergic gliotransmission: a modeling study. Neural Plast. 2016:7607924. 10.1155/2016/7607924 27195153 PMC4852535

[B40] De Pittà M. Brunel N. Volterra A. (2016). Astrocytes: orchestrating synaptic plasticity? Neuroscience 323, 43–61. 10.1016/j.neuroscience.2015.04.001 25862587

[B41] De Pittà M. Goldberg M. Volman V. Berry H. Ben-Jacob E. (2009a). Glutamate regulation of calcium and IP_3_ oscillating and pulsating dynamics in astrocytes. J. Biol. Phys. 35, 383–411. 10.1007/s10867-009-9155-y 19669422 PMC2750743

[B42] De Pittà M. Volman V. Berry H. Parpura V. Volterra A. Ben-Jacob E. (2012). Computational quest for understanding the role of astrocyte signaling in synaptic transmission and plasticity. Front. Comput. Neurosci. 6:98. 10.3389/fncom.2012.00098 23267326 PMC3528083

[B43] De Pittà M. Volman V. Levine H. Ben-Jacob E. (2009b). Multimodal encoding in a simplified model of intracellular calcium signaling. Cogn. Process. 10(Suppl. 1), S55–S70. 10.1007/s10339-008-0242-y 19030904

[B44] De Schutter E. (2008). Why are computational neuroscience and systems biology so separate. PLoS Comput. Biol. 4:e1000078. 10.1371/journal.pcbi.1000078 18516226 PMC2367448

[B45] De Young G. W. Keizer J. (1992). A single-pool inositol 1,4,5-trisphosphate-receptor-based model for agonist-stimulated oscillations in Ca^2+^ concentration. Proc. Natl. Acad. Sci. U.S.A. 89, 9895–9899. 10.1073/pnas.89.20.9895 1329108 PMC50240

[B46] Di Garbo A. (2009). Dynamics of a minimal neural model consisting of an astrocyte, a neuron, and an interneuron. J. Biol. Phys. 35, 361–382. 10.1007/s10867-009-9143-2 19669428 PMC2750740

[B47] Di Garbo A. Barbi M. Chillemi S. Alloisio S. Nobile M. (2007). Calcium signalling in astrocytes and modulation of neural activity. Biosystems 89, 74–83. 10.1016/j.biosystems.2006.05.013 17196325

[B48] Diekman C. O. Fall C. P. Lechleiter J. D. Terman D. (2013). Modeling the neuroprotective role of enhanced astrocyte mitochondrial metabolism during stroke. Biophys. J. 104, 1752–1763. 10.1016/j.bpj.2013.02.025 23601322 PMC3627876

[B49] Ding F. O'Donnell J. Thrane A. S. Zeppenfeld D. Kang H. Xie L. Wang F. Nedergaard M. (2013). α_1_-Adrenergic receptors mediate coordinated Ca^2+^ signaling of cortical astrocytes in awake, behaving mice. Cell Calcium 54, 387–394. 10.1016/j.ceca.2013.09.001 24138901 PMC3858490

[B50] Ding X. Zhang X. Ji L. (2018). Contribution of calcium fluxes to astrocyte spontaneous calcium oscillations in deterministic and stochastic models. Appl. Math. Model. 55, 371–382. 10.1016/j.apm.2017.11.002

[B51] DiNuzzo M. Gili T. Maraviglia B. Giove F. (2011). Modeling the contribution of neuron-astrocyte cross talk to slow blood oxygenation level-dependent signal oscillations. J. Neurophysiol. 106, 3010–3018. 10.1152/jn.00416.2011 21917999

[B52] Dupont G. Falcke M. Kirk V. Sneyd J. (2016). Models of Calcium Signalling, Vol. 43 Springer.

[B53] Dupont G. Lokenye E. F. L. Challiss R. A. J. (2011). A model for Ca^2+^ oscillations stimulated by the type 5 metabotropic glutamate receptor: an unusual mechanism based on repetitive, reversible phosphorylation of the receptor. Biochimie 93, 2132–2138. 10.1016/j.biochi.2011.09.010 21945596

[B54] Edwards J. R. Gibson W. G. (2010). A model for Ca^2+^ waves in networks of glial cells incorporating both intercellular and extracellular communication pathways. J. Theor. Biol. 263, 45–58. 10.1016/j.jtbi.2009.12.002 20005235

[B55] Ermentrout B. (2002). Simulating, Analyzing, and Animating Dynamical Systems: A Guide to XPPAUT for Researchers and Students, 1st Edn. Philadelphia, PA: Society for Industrial & Applied Mathematics (SIAM).

[B56] Farr H. David T. (2011). Models of neurovascular coupling via potassium and EET signalling. J. Theor. Biol. 286, 13–23. 10.1016/j.jtbi.2011.07.006 21781976

[B57] Fellin T. Ellenbogen J. M. De Pittà M. Ben-Jacob E. Halassa M. M. (2012). Astrocyte regulation of sleep circuits: experimental and modeling perspectives. Front. Comput. Neurosci. 6:65. 10.3389/fncom.2012.00065 22973222 PMC3428699

[B58] Fellin T. Pascual O. Haydon P. G. (2006). Astrocytes coordinate synaptic networks: balanced excitation and inhibition. Physiology 21, 208–215. 10.1152/physiol.00161.2005 16714479

[B59] Fiacco T. A. Agulhon C. Taves S. R. Petravicz J. Casper K. B. Dong X. (2007). Selective stimulation of astrocyte calcium in situ does not affect neuronal excitatory synaptic activity. Neuron 54, 611–626. 10.1016/j.neuron.2007.04.032 17521573

[B60] Fiacco T. A. McCarthy K. D. (2018). Multiple lines of evidence indicate that gliotransmission does not occur under physiological conditions. J. Neurosci. 38, 3–13. 10.1523/JNEUROSCI.0016-17.2017 29298904 PMC5761435

[B61] FitzHugh R. (1961). Impulses and physiological states in theoretical models of nerve membrane. Biophys. J. 1, 445–466. 10.1016/S0006-3495(61)86902-6 19431309 PMC1366333

[B62] Freeman M. R. (2010). Specification and morphogenesis of astrocytes. Science 330, 774–778. 10.1126/science.1190928 21051628 PMC5201129

[B63] Fujita T. Chen M. J. Li B. Smith N. A. Peng W. Sun W.. (2014). Neuronal transgene expression in dominant-negative SNARE mice. J. Neurosci. 34, 16594–16604. 10.1523/JNEUROSCI.2585-14.2014 25505312 PMC4261088

[B64] Gerstner W. Kistler W. M. (2002). Spiking Neuron Models: Single Neurons, Populations, Plasticity. Cambridge, UK: Cambridge University Press.

[B65] Ghosh S. Kaushik D. K. Gomes J. Nayeem S. Deep S. Basu A. (2010). Changes in cytosolic Ca^2+^ levels correspond to fluctuations of lactate levels in crosstalk of astrocyte-neuron cell lines. Indian J. Exp. Biol. 48, 529–537. 20882752

[B66] Giaume C. Venance L. (1998). Intercellular calcium signaling and gap junctional communication in astrocytes. Glia 24, 50–64. 10.1002/(SICI)1098-1136(199809)24:1<50::AID-GLIA6>3.0.CO;2-4 9700489

[B67] Gibson W. G. Farnell L. Bennett M. R. (2007). A computational model relating changes in cerebral blood volume to synaptic activity in neurons. Neurocomputing 70, 1674–1679. 10.1016/j.neucom.2006.10.071

[B68] Gibson W. G. Farnell L. Bennett M. R. (2008). A quantitative model of ATP-mediated calcium wave propagation in astrocyte networks, in Mathematical Modeling of Biological Systems, Vol. II, eds Deutsch A. Bravo de la Parra R. de Boer R. J. Diekmann O. Jagers P. Kisdi E. Kretzschmar M. Lansky P. Metz H. (Boston, MA: Birkhäuser), 193–204.

[B69] Gillespie D. T. (1976). A general method for numerically simulating the stochastic time evolution of coupled chemical reactions. J. Comput. Phys. 22, 403–434. 10.1016/0021-9991(76)90041-3

[B70] Gillespie D. T. (1977). Exact stochastic simulation of coupled chemical reactions. J. Phys. Chem. 81, 2340–2361. 10.1021/j100540a008

[B71] Gillespie D. T. (2000). The chemical Langevin equation. J. Chem. Phys. 113, 297–306. 10.1063/1.481811

[B72] Gillespie D. T. (2001). Approximate accelerated stochastic simulation of chemically reacting systems. J. Chem. Phys. 115, 1716–1733. 10.1063/1.1378322

[B73] Glaum S. R. Holzwarth J. A. Miller R. J. (1990). Glutamate receptors activate Ca^2+^ mobilization and Ca^2+^ influx into astrocytes. Proc. Natl. Acad. Sci. U.S.A. 87, 3454–3458. 10.1073/pnas.87.9.3454 1970637 PMC53919

[B74] Goldberg M. De Pittà M. Volman V. Berry H. Ben-Jacob E. (2010). Nonlinear gap junctions enable long-distance propagation of pulsating calcium waves in astrocyte networks. PLoS Comput. Biol. 6:e1000909. 10.1371/journal.pcbi.1000909 20865153 PMC2928752

[B75] Goto I. Kinoshita S. Natsume K. (2004). The model of glutamate-induced intracellular Ca^2+^ oscillation and intercellular Ca^2+^ wave in brain astrocytes. Neurocomputing 58–60, 461–467. 10.1016/j.neucom.2004.01.082

[B76] Grillner S. Ip N. Koch C. Koroshetz W. Okano H. Polachek M.. (2016). Worldwide initiatives to advance brain research. Nat. Neurosci. 19, 1118–1122. 10.1038/nn.4371 27571190 PMC6047900

[B77] Guo S. Tang J. Ma J. Wang C. (2017). Autaptic modulation of electrical activity in a network of neuron-coupled astrocyte. Complexity 2017:4631602 10.1155/2017/4631602

[B78] Guthrie P. B. Knappenberger J. Segal M. Bennett M. V. L. Charles A. C. Kater S. B. (1999). ATP released from astrocytes mediates glial calcium waves. J. Neurosci. 19, 520–528. 9880572 10.1523/JNEUROSCI.19-02-00520.1999 PMC6782195

[B79] Hadfield J. Plank M. J. David T. (2013). Modeling secondary messenger pathways in neurovascular coupling. Bull. Math. Biol. 75, 428–443. 10.1007/s11538-013-9813-x 23358799

[B80] Haghiri S. Ahmadi A. Saif M. (2016). VLSI implementable neuron-astrocyte control mechanism. Neurocomputing 214, 280–296. 10.1016/j.neucom.2016.06.015

[B81] Haghiri S. Ahmadi A. Saif M. (2017). Complete neuron-astrocyte interaction model: digital multiplierless design and networking mechanism. IEEE Trans. Biomed. Circuits Syst. 11, 117–127. 10.1109/TBCAS.2016.2583920 27662685

[B82] Haim L. B. Rowitch D. H. (2017). Functional diversity of astrocytes in neural circuit regulation. Nat. Rev. Neurosci. 18, 31–41. 10.1038/nrn.2016.159 27904142

[B83] Halassa M. M. Fellin T. Haydon P. G. (2007). The tripartite synapse: roles for gliotransmission in health and disease. Trends Mol. Med. 13, 54–63. 10.1016/j.molmed.2006.12.005 17207662

[B84] Halassa M. M. Haydon P. G. (2010). Integrated brain circuits: astrocytic networks modulate neuronal activity and behavior. Annu. Rev. Physiol. 72, 335–355. 10.1146/annurev-physiol-021909-135843 20148679 PMC3117429

[B85] Hamilton N. B. Attwell D. (2010). Do astrocytes really exocytose neurotransmitters? Nat. Rev. Neurosci. 11, 227–238. 10.1038/nrn2803 20300101

[B86] Handy G. Taheri M. White J. A. Borisyuk A. (2017). Mathematical investigation of IP_3_-dependent calcium dynamics in astrocytes. J. Comput. Neurosci. 42, 257–273. 10.1007/s10827-017-0640-1 28353176 PMC5756620

[B87] Hayati M. Nouri M. Haghiri S. Abbott D. (2016). A digital realization of astrocyte and neural glial interactions. IEEE Trans. Biomed. Circuits Syst. 10, 518–529. 10.1109/TBCAS.2015.2450837 26390499

[B88] Henneberger C. Papouin T. Oliet S. H. R. Rusakov D. A. (2010). Long-term potentiation depends on release of D-serine from astrocytes. Nature 463, 232–236. 10.1038/nature08673 20075918 PMC2807667

[B89] Hepburn I. Chen W. Wils S. De Schutter E. (2012). STEPS: efficient simulation of stochastic reaction-diffusion models in realistic morphologies. BMC Syst. Biol. 6:36. 10.1186/1752-0509-6-36 22574658 PMC3472240

[B90] Hirase H. Qian L. Barthó P. Buzsáki G. (2004). Calcium dynamics of cortical astrocytic networks *in vivo*. PLoS Biol. 2:e96. 10.1371/journal.pbio.0020096 15094801 PMC387267

[B91] Hodgkin A. L. Huxley A. F. (1952). A quantitative description of membrane current and its application to conduction and excitation in nerve. J. Physiol. 117, 500–544. 10.1113/jphysiol.1952.sp004764 12991237 PMC1392413

[B92] Höfer T. Venance L. Giaume C. (2002). Control and plasticity of intercellular calcium waves in astrocytes: a modeling approach. J. Neurosci. 22, 4850–4859. 12077182 10.1523/JNEUROSCI.22-12-04850.2002 PMC6757753

[B93] Houart G. Dupont G. Goldbeter A. (1999). Bursting, chaos and birhythmicity originating from self-modulation of the inositol 1,4,5-trisphosphate signal in a model for intracellular Ca^2+^ oscillations. Bull. Math. Biol. 61, 507–530. 10.1006/bulm.1999.0095 17883229

[B94] Iacobas D. A. Suadicani S. O. Spray D. C. Scemes E. (2006). A stochastic two-dimensional model of intercellular Ca^2+^ wave spread in glia. Biophys. J. 90, 24–41. 10.1529/biophysj.105.064378 16214872 PMC1367023

[B95] Izhikevich E. M. (2007). Dynamical Systems in Neuroscience. Cambridge, MA: The MIT Press.

[B96] Jalonen T. O. Margraf R. R. Wielt D. B. Charniga C. J. Linne M.-L. Kimelberg H. K. (1997). Serotonin induces inward potassium and calcium currents in rat cortical astrocytes. Brain Res. 758, 69–82. 10.1016/S0006-8993(97)00163-7 9203535

[B97] Jolivet R. Allaman I. Pellerin L. Magistretti P. J. Weber B. (2010). Comment on recent modeling studies of astrocyte–neuron metabolic interactions. J. Cereb. Blood Flow Metab. 30, 1982–1986. 10.1038/jcbfm.2010.132 20700131 PMC3002878

[B98] Kang M. Othmer H. G. (2009). Spatiotemporal characteristics of calcium dynamics in astrocytes. Chaos 19:037116. 10.1063/1.3206698 19792041 PMC2852438

[B99] Karimi G. Ranjbar M. Amirian M. Shahim-aeen A. (2018). A neuromorphic real-time VLSI design of Ca^2+^ dynamic in an astrocyte. Neurocomputing 272, 197–203. 10.1016/j.neucom.2017.06.071

[B100] Kazantsev V. B. (2009). Spontaneous calcium signals induced by gap junctions in a network model of astrocytes. Phys. Rev. E 79:010901. 10.1103/PhysRevE.79.010901 19256994

[B101] Keener J. Sneyd J. (1998). Mathematical Physiology. New York, NY: Springer-Verlag.

[B102] Keener J. Sneyd J. (2009). Mathematical Physiology: I: Cellular Physiology. New York, NY: Springer.

[B103] Kenny A. Plank M. J. David T. (2018). The role of astrocytic calcium and TRPV4 channels in neurovascular coupling. J. Comput. Neurosci. 44, 97–114. 10.1007/s10827-017-0671-7 29152668

[B104] Kerr R. A. Bartol T. M. Kaminsky B. Dittrich M. Chang J.-C. J. Baden S. B.. (2008). Fast Monte Carlo simulation methods for biological reaction-diffusion systems in solution and on surfaces. SIAM J. Sci. Comput. 30, 3126–3149. 10.1137/070692017 20151023 PMC2819163

[B105] Kettenmann H. Ransom B. R. (eds). (2013). Neuroglia, 3rd Edn. New York, NY: Oxford University Press.

[B106] Khakh B. S. Sofroniew M. V. (2015). Diversity of astrocyte functions and phenotypes in neural circuits. Nat. Neurosci. 18, 942–952. 10.1038/nn.4043 26108722 PMC5258184

[B107] Kimelberg H. K. (1995). Receptors on astrocytes-what possible functions? Neurochem. Int. 26, 27–40. 10.1016/0197-0186(94)00118-E 7787760

[B108] Komin N. Moein M. Ellisman M. H. Skupin A. (2015). Multiscale modeling indicates that temperature dependent [Ca^2+^]_i_ spiking in astrocytes is quantitatively consistent with modulated SERCA activity. Neural Plast. 2015:683490. 10.1155/2015/683490 26347125 PMC4539483

[B109] Lallouette J. De Pittà M. Ben-Jacob E. Berry H. (2014). Sparse short-distance connections enhance calcium wave propagation in a 3D model of astrocyte networks. Front. Comput. Neurosci. 8:45. 10.3389/fncom.2014.00045 24795613 PMC3997029

[B110] Larter R. Craig M. G. (2005). Glutamate-induced glutamate release: a proposed mechanism for calcium bursting in astrocytes. Chaos 15, 047511. 10.1063/1.2102467 16396604

[B111] Lavrentovich M. Hemkin S. (2008). A mathematical model of spontaneous calcium (II) oscillations in astrocytes. J. Theor. Biol. 251, 553–560. 10.1016/j.jtbi.2007.12.011 18275973

[B112] Lecca P. Bagagiolo F. Scarpa M. (2017). Hybrid deterministic/stochastic simulation of complex biochemical systems. Mol. BioSyst. 13, 2672–2686. 10.1039/C7MB00426E 29058744

[B113] Lehtimäki M. Paunonen L. Pohjolainen S. Linne M.-L. (2017). Order reduction for a signaling pathway model of neuronal synaptic plasticity. IFAC-PapersOnLine 50, 7687–7692. 10.1016/j.ifacol.2017.08.1143

[B114] Li B. Chen S. Zeng S. Luo Q. Li P. (2012). Modeling the contributions of Ca^2+^ flows to spontaneous Ca^2+^ oscillations and cortical spreading depression-triggered Ca^2+^ waves in astrocyte networks. PLoS ONE 7:e48534. 10.1371/journal.pone.0048534 23119049 PMC3485305

[B115] Li D. Agulhon C. Schmidt E. Oheim M. Ropert N. (2013). New tools for investigating astrocyte-to-neuron communication. Front. Cell. Neurosci. 7:193. 10.3389/fncel.2013.00193 24194698 PMC3810613

[B116] Li J. Tang J. Ma J. Du M. Wang R. Wu Y. (2016a). Dynamic transition of neuronal firing induced by abnormal astrocytic glutamate oscillation. Sci. Rep. 6:32343. 10.1038/srep32343 27573570 PMC5004107

[B117] Li J. Wang R. Du M. Tang J. Wu Y. (2016b). Dynamic transition on the seizure-like neuronal activity by astrocytic calcium channel block. Chaos Soliton. Fract. 91, 702–708. 10.1016/j.chaos.2016.08.009

[B118] Li J. Du M.-M. Wang R. Lei J.-Z. Wu Y. (2016c). Astrocytic gliotransmitter: diffusion dynamics and induction of information processing on tripartite synapses. Int. J. Bifurcat. Chaos 26:1650138 10.1142/S0218127416501388

[B119] Li J. J. Xie Y. Yu Y. G. Du M. M. Wang R. Wu Y. (2017). A neglected GABAergic astrocyte: calcium dynamics and involvement in seizure activity. Sci. China Tech. Sci. 60, 1003–1010. 10.1007/s11431-016-9056-2

[B120] Li Y.-X. Rinzel J. (1994). Equations for InsP_3_ receptor-mediated [Ca^2+^]_i_ oscillations derived from a detailed kinetic model: a Hodgkin-Huxley like formalism. J. Theor. Biol. 166, 461–473. 10.1006/jtbi.1994.1041 8176949

[B121] Linne M.-L. Jalonen T. O. (2014). Astrocyte–neuron interactions: from experimental research-based models to translational medicine. Prog. Mol. Biol. Transl. Sci. 123, 191–217. 10.1016/B978-0-12-397897-4.00005-X 24560146

[B122] Liu J. Harkin J. Maguire L. P. McDaid L. J. Wade J. J. (2018). SPANNER: a self-repairing spiking neural network hardware architecture. IEEE Trans. Neural Netw. Learn. Syst. 29, 1287–1300. 10.1109/TNNLS.2017.2673021 28287992

[B123] Liu J. Harkin J. Maguire L. P. McDaid L. J. Wade J. J. Martin G. (2016). Scalable networks-on-chip interconnected architecture for astrocyte-neuron networks. IEEE Trans. Circuits Syst. I Reg. Papers 63, 2290–2303. 10.1109/TCSI.2016.2615051

[B124] Liu Y. Li C. (2013a). Firing rate propagation through neuronal–astrocytic network. IEEE Trans. Neural Netw. Learn. Syst. 24, 789–799. 10.1109/TNNLS.2013.2245678 24808428

[B125] Liu Y. Li C. (2013b). Stochastic resonance in feedforward-loop neuronal network motifs in astrocyte field. J. Theor. Biol. 335, 265–275. 10.1016/j.jtbi.2013.07.007 23871712

[B126] López-Caamal F. Oyarzún D. A. Middleton R. H. García M. R. (2014). Spatial quantification of cytosolic Ca^2+^ accumulation in nonexcitable cells: an analytical study. IEEE/ACM Trans. Comput. Biol. Bioinform. 11, 592–603. 10.1109/TCBB.2014.2316010 26356026

[B127] Ma Z. Stork T. Bergles D. E. Freeman M. R. (2016). Neuromodulators signal through astrocytes to alter neural circuit activity and behaviour. Nature 539, 428–432. 10.1038/nature20145 27828941 PMC5161596

[B128] MacDonald C. L. Silva G. A. (2013). A positive feedback cell signaling nucleation model of astrocyte dynamics. Front. Neuroeng. 6:4. 10.3389/fneng.2013.00004 23847529 PMC3706728

[B129] Magistretti P. J. Allaman I. (2015). A cellular perspective on brain energy metabolism and functional imaging. Neuron 86, 883–901. 10.1016/j.neuron.2015.03.035 25996133

[B130] Mangia S. DiNuzzo M. Giove F. Carruthers A. Simpson I. A. Vannucci S. J. (2011). Response to ‘comment on recent modeling studies of astrocyte–neuron metabolic interactions’: Much ado about nothing. J. Cereb. Blood Flow Metab. 31, 1346–1353. 10.1038/jcbfm.2011.29 21427731 PMC3130323

[B131] Manninen T. Havela R. Linne M.-L. (2017). Reproducibility and comparability of computational models for astrocyte calcium excitability. Front. Neuroinform. 11:11. 10.3389/fninf.2017.00011 28270761 PMC5318440

[B132] Manninen T. Havela R. Linne M.-L. (2018). Computational models of astrocytes and astrocyte-neuron interactions: characterization, reproducibility, and future perspectives, in Mathematical Methods in Modeling of Neuron-Glia Interactions, eds De Pittà M. Berry H. (Springer).

[B133] Manninen T. Hituri K. Hellgren Kotaleski J. Blackwell K. T. Linne M.-L. (2010). Postsynaptic signal transduction models for long-term potentiation and depression. Front. Comput. Neurosci. 4:152. 10.3389/fncom.2010.00152 21188161 PMC3006457

[B134] Manninen T. Linne M.-L. Ruohonen K. (2006a). Developing Itô stochastic differential equation models for neuronal signal transduction pathways. Comput. Biol. Chem. 30, 280–291. 10.1016/j.compbiolchem.2006.04.002 16880117

[B135] Manninen T. Linne M.-L. Ruohonen K. (2006b). A novel approach to model neuronal signal transduction using stochastic differential equations. Neurocomputing 69, 1066–1069. 10.1016/j.neucom.2005.12.047 16880117

[B136] Markram H. Muller E. Ramaswamy S. Reimann M. W. Abdellah M. Sanchez C. A.. (2015). Reconstruction and simulation of neocortical microcircuitry. Cell 163, 456–492. 10.1016/j.cell.2015.09.029 26451489

[B137] Matrosov V. V. Kazantsev V. B. (2011). Bifurcation mechanisms of regular and chaotic network signaling in brain astrocytes. Chaos 21:023103. 10.1063/1.3574031 21721745

[B138] McDougal R. A. Bulanova A. S. Lytton W. W. (2016). Reproducibility in computational neuroscience models and simulations. IEEE Trans. Biomed. Eng. 63, 2021–2035. 10.1109/TBME.2016.2539602 27046845 PMC5016202

[B139] Mesiti F. Floor P. A. Balasingham I. (2015a). Astrocyte to neuron communication channels with applications. IEEE Trans. Mol. Biol. Multi-Scale Commun. 1, 164–175. 10.1109/TMBMC.2015.2501743

[B140] Mesiti F. Veletić M. Floor P. A. Balasingham I. (2015b). Astrocyte–neuron communication as cascade of equivalent circuits. Nano Commun. Netw. 6, 183–197. 10.1016/j.nancom.2015.08.005

[B141] Min R. Santello M. Nevian T. (2012). The computational power of astrocyte mediated synaptic plasticity. Front. Comput. Neurosci. 6:93. 10.3389/fncom.2012.00093 23125832 PMC3485583

[B142] Montaseri G. Yazdanpanah M. J. (2014). Desynchronization of two coupled limit-cycle oscillators using an astrocyte-inspired controller. Int. J. Biomath. 7:1450001 10.1142/S1793524514500016

[B143] Morris C. Lecar H. (1981). Voltage oscillations in the barnacle giant muscle fiber. Biophys. J. 35, 193–213. 10.1016/S0006-3495(81)84782-0 7260316 PMC1327511

[B144] Munafò M. R. Nosek B. A. Bishop D. V. M. Button K. S. Chambers C. D. du Sert N. P. (2017). A manifesto for reproducible science. Nat. Hum. Behav. 1:0021 10.1038/s41562-016-0021 PMC7610724 33954258

[B145] Nadkarni S. Jung P. (2003). Spontaneous oscillations of dressed neurons: a new mechanism for epilepsy? Phys. Rev. Lett. 91:268101. 10.1103/PhysRevLett.91.268101 14754091

[B146] Nadkarni S. Jung P. (2004). Dressed neurons: modeling neural–glial interactions. Phys. Biol. 1, 35. 10.1088/1478-3967/1/1/004 16204820

[B147] Nadkarni S. Jung P. (2005). Synaptic inhibition and pathologic hyperexcitability through enhanced neuron-astrocyte interaction: a modeling study. J. Integr. Neurosci. 4, 207–226. 10.1142/S0219635205000811 15988798

[B148] Nadkarni S. Jung P. (2007). Modeling synaptic transmission of the tripartite synapse. Phys. Biol., 4, 1–9. 10.1088/1478-3975/4/1/001 17406080

[B149] Nadkarni S. Jung P. Levine H. (2008). Astrocytes optimize the synaptic transmission of information. PLoS Comput. Biol. 4:e1000088. 10.1371/journal.pcbi.1000088 18516277 PMC2390854

[B150] Naeem M. McDaid L. J. Harkin J. Wade J. J. Marsland J. (2015). On the role of astroglial syncytia in self-repairing spiking neural networks. IEEE Trans. Neural Netw. Learn. Syst. 26, 2370–2380. 10.1109/TNNLS.2014.2382334 25576582

[B151] Nazari S. Amiri M. Faez K. Amiri M. (2015a). Multiplier-less digital implementation of neuron–astrocyte signalling on FPGA. Neurocomputing 164, 281–292. 10.1016/j.neucom.2015.02.041

[B152] Nazari S. Faez K. Amiri M. (2017). A multiplier-less digital design of a bio-inspired stimulator to suppress synchronized regime in a large-scale, sparsely connected neural network. Neural Comput. Appl. 28, 375–390. 10.1007/s00521-015-2071-0

[B153] Nazari S. Faez K. Amiri M. Karami E. (2015b). A digital implementation of neuron–astrocyte interaction for neuromorphic applications. Neural Netw. 66, 79–90. 10.1016/j.neunet.2015.01.005 25814323

[B154] Nazari S. Faez K. Amiri M. Karami E. (2015c). A novel digital implementation of neuron–astrocyte interactions. J. Comput. Electron. 14, 227–239. 10.1007/s10825-014-0643-3 25814323

[B155] Nedergaard M. (1994). Direct signaling from astrocytes to neurons in cultures of mammalian brain cells. Science 263, 1768–1771. 10.1126/science.8134839 8134839

[B156] Newman E. A. Zahs K. R. (1997). Calcium waves in retinal glial cells. Science 275, 844–847. 10.1126/science.275.5301.844 9012354 PMC2410141

[B157] Nimmerjahn A. (2009). Astrocytes going live: advances and challenges. J. Physiol. 587, 1639–1647. 10.1113/jphysiol.2008.167171 19204050 PMC2683952

[B158] Nimmerjahn A. Mukamel E. A. Schnitzer M. J. (2009). Motor behavior activates Bergmann glial networks. Neuron 62, 400–412. 10.1016/j.neuron.2009.03.019 19447095 PMC2820366

[B159] Nordlie E. Gewaltig M.-O. Plesser H. E. (2009). Towards reproducible descriptions of neuronal network models. PLoS Comput. Biol. 5:e1000456. 10.1371/journal.pcbi.1000456 19662159 PMC2713426

[B160] Oberheim N. A. Takano T. Han X. He W. Lin J. H. C. Wang F.. (2009). Uniquely hominid features of adult human astrocytes. J. Neurosci. 29, 3276–3287. 10.1523/JNEUROSCI.4707-08.2009 19279265 PMC2819812

[B161] Oku Y. Fresemann J. Miwakeichi F. Hülsmann S. (2016). Respiratory calcium fluctuations in low-frequency oscillating astrocytes in the pre-Bötzinger complex. Respir. Physiol. Neurobiol. 226, 11–17. 10.1016/j.resp.2015.02.002 25747384

[B162] Oliveira R. F. Terrin A. Di Benedetto G. Cannon R. C. Koh W. Kim M.. (2010). The role of type 4 phosphodiesterases in generating microdomains of cAMP: large scale stochastic simulations. PLoS ONE 5:e11725. 10.1371/journal.pone.0011725 20661441 PMC2908681

[B163] Olude M. A. Mustapha O. A. Aderounmu O. A. Olopade J. O. Ihunwo A. O. (2015). Astrocyte morphology, heterogeneity, and density in the developing African giant rat (Cricetomys gambianus). Front. Neuroanat. 9:67. 10.3389/fnana.2015.00067 26074782 PMC4443027

[B164] Olufsen M. S. Whittington M. A. Camperi M. Kopell N. (2003). New roles for the gamma rhythm: population tuning and preprocessing for the beta rhythm. J. Comput. Neurosci. 14, 33–54. 10.1023/A:1021124317706 12435923

[B165] Oschmann F. Mergenthaler K. Jungnickel E. Obermayer K. (2017). Spatial separation of two different pathways accounting for the generation of calcium signals in astrocytes. PLoS Comput. Biol. 13:e1005377. 10.1371/journal.pcbi.1005377 28192424 PMC5330534

[B166] Otsu Y. Couchman K. Lyons D. G. Collot M. Agarwal A. Mallet J.-M.. (2015). Calcium dynamics in astrocyte processes during neurovascular coupling. Nat. Neurosci. 18, 210–218. 10.1038/nn.3906 25531572 PMC4651918

[B167] Parpura V. Basarsky T. A. Liu F. Jeftinija K. Jeftinija S. Haydon P. G. (1994). Glutamate-mediated astrocyte–neuron signalling. Nature 369, 744–747. 10.1038/369744a0 7911978

[B168] Parri H. R. Gould T. M. Crunelli V. (2001). Spontaneous astrocytic Ca^2+^ oscillations in situ drive NMDAR-mediated neuronal excitation. Nat. Neurosci. 4, 803–812. 10.1038/90507 11477426

[B169] Patrushev I. Gavrilov N. Turlapov V. Semyanov A. (2013). Subcellular location of astrocytic calcium stores favors extrasynaptic neuron–astrocyte communication. Cell Calcium 54, 343–349. 10.1016/j.ceca.2013.08.003 24035346

[B170] Paukert M. Agarwal A. Cha J. Doze V. A. Kang J. U. Bergles D. E. (2014). Norepinephrine controls astroglial responsiveness to local circuit activity. Neuron 82, 1263–1270. 10.1016/j.neuron.2014.04.038 24945771 PMC4080721

[B171] Perc M. Green A. K. Dixon C. J. Marhl M. (2008). Establishing the stochastic nature of intracellular calcium oscillations from experimental data. Biophys. Chem. 132, 33–38. 10.1016/j.bpc.2007.10.002 17964062

[B172] Perea G. Navarrete M. Araque A. (2009). Tripartite synapses: astrocytes process and control synaptic information. Trends Neurosci. 32, 421–431. 10.1016/j.tins.2009.05.001 19615761

[B173] Peters O. Schipke C. G. Hashimoto Y. Kettenmann H. (2003). Different mechanisms promote astrocyte Ca^2+^ waves and spreading depression in the mouse neocortex. J. Neurosci. 23, 9888–9896. 14586018 10.1523/JNEUROSCI.23-30-09888.2003 PMC6740882

[B174] Petravicz J. Fiacco T. A. McCarthy K. D. (2008). Loss of IP_3_ receptor-dependent Ca^2+^ increases in hippocampal astrocytes does not affect baseline CA1 pyramidal neuron synaptic activity. J. Neurosci. 28, 4967–4973. 10.1523/JNEUROSCI.5572-07.2008 18463250 PMC2709811

[B175] Pinsky P. F. Rinzel J. (1994). Intrinsic and network rhythmogenesis in a reduced Traub model for CA3 neurons. J. Comput. Neurosci. 1, 39–60. 10.1007/BF00962717 8792224

[B176] Porter J. T. McCarthy K. D. (1996). Hippocampal astrocytes *in situ* respond to glutamate released from synaptic terminals. J. Neurosci. 16, 5073–5081. 8756437 10.1523/JNEUROSCI.16-16-05073.1996 PMC6579292

[B177] Poskanzer K. E. Yuste R. (2016). Astrocytes regulate cortical state switching in vivo. Proc. Natl. Acad. Sci. U.S.A. 113, E2675–E2684. 10.1073/pnas.1520759113 27122314 PMC4868485

[B178] Postnov D. E. Koreshkov R. N. Brazhe N. A. Brazhe A. R. Sosnovtseva O. V. (2009). Dynamical patterns of calcium signaling in a functional model of neuron-astrocyte networks. J. Biol. Phys., 35, 425–445. 10.1007/s10867-009-9156-x 19669421 PMC2750744

[B179] Postnov D. E. Ryazanova L. S. Sosnovtseva O. V. (2007). Functional modeling of neural-glial interaction. BioSystems 89, 84–91. 10.1016/j.biosystems.2006.04.012 17320272

[B180] Rao C. V. Wolf D. M. Arkin A. P. (2002). Control, exploitation and tolerance of intracellular noise. Nature 420, 231–237. 10.1038/nature01258 12432408

[B181] Raser J. M. O'Shea E. K. (2005). Noise in gene expression: origins, consequences, and control. Science 309, 2010–2013. 10.1126/science.1105891 16179466 PMC1360161

[B182] Ribrault C. Sekimoto K. Triller A. (2011). From the stochasticity of molecular processes to the variability of synaptic transmission. Nat. Rev. Neurosci. 12, 375–387. 10.1038/nrn3025 21685931

[B183] Riera J. Hatanaka R. Ozaki T. Kawashima R. (2011a). Modeling the spontaneous Ca^2+^ oscillations in astrocytes: inconsistencies and usefulness. J. Integr. Neurosci., 10, 439–473. 10.1142/S0219635211002877 22262535

[B184] Riera J. Hatanaka R. Uchida T. Ozaki T. Kawashima R. (2011b). Quantifying the uncertainty of spontaneous Ca^2+^ oscillations in astrocytes: particulars of Alzheimer's disease. Biophys. J. 101, 554–564. 10.1016/j.bpj.2011.06.041 21806923 PMC3145292

[B185] Roth B. J. Yagodin S. V. Holtzclaw L. Russell J. T. (1995). A mathematical model of agonist-induced propagation of calcium waves in astrocytes. Cell Calcium 17, 53–64. 10.1016/0143-4160(95)90102-7 7553781

[B186] Rougier N. P. Hinsen K. Alexandre F. Arildsen T. Barba L. A. Benureau F. C. Y. (2017). Sustainable computational science: the ReScience initiative. PeerJ Comput. Sci. 3:e142 10.7717/peerj-cs.142 PMC8530091 34722870

[B187] Salis H. Sotiropoulos V. Kaznessis Y. N. (2006). Multiscale Hy3S: hybrid stochastic simulation for supercomputers. BMC Bioinformatics 7:93. 10.1186/1471-2105-7-93 16504125 PMC1421438

[B188] Savtchouk I. Volterra A. (2018). Gliotransmission: beyond black-and-white. J. Neurosci. 38, 14–25. 10.1523/JNEUROSCI.0017-17.2017 29298905 PMC6705815

[B189] Shuai J.-W. Jung P. (2002). Stochastic properties of Ca^2+^ release of inositol 1, 4, 5-trisphosphate receptor clusters. Biophys. J. 83, 87–97. 10.1016/S0006-3495(02)75151-5 12080102 PMC1302129

[B190] Sibille J. Pannasch U. Rouach N. (2014). Astroglial potassium clearance contributes to short-term plasticity of synaptically evoked currents at the tripartite synapse. J. Physiol. 592, 87–102. 10.1113/jphysiol.2013.261735 24081156 PMC3903353

[B191] Silchenko A. N. Tass P. A. (2008). Computational modeling of paroxysmal depolarization shifts in neurons induced by the glutamate release from astrocytes. Biol. Cybern. 98, 61–74. 10.1007/s00422-007-0196-7 18064484

[B192] Skupin A. Kettenmann H. Falcke M. (2010). Calcium signals driven by single channel noise. PLoS Comput. Biol. 6:e1000870. 10.1371/journal.pcbi.1000870 20700497 PMC2917103

[B193] Skupin A. Kettenmann H. Winkler U. Wartenberg M. Sauer H. Tovey S. C. (2008). How does intracellular Ca^2+^ oscillate: by chance or by the clock? Biophys. J. 94, 2404–2411. 10.1529/biophysj.107.119495 18065468 PMC2257893

[B194] Sloan S. A. Barres B. A. (2014). Looks can be deceiving: reconsidering the evidence for gliotransmission. Neuron 84, 1112–1115. 10.1016/j.neuron.2014.12.003 25521372 PMC4433290

[B195] Sneyd J. Charles A. C. Sanderson M. J. (1994). A model for the propagation of intercellular calcium waves. Am. J. Physiol., Cell Physiol. 266, C293–C302. 10.1152/ajpcell.1994.266.1.C293 8304425

[B196] Soleimani H. Bavandpour M. Ahmadi A. Abbott D. (2015). Digital implementation of a biological astrocyte model and its application. IEEE Trans. Neural Netw. Learn. Syst. 26, 127–139. 10.1109/TNNLS.2014.2311839 25532161

[B197] Sompolinsky H. (2014). Computational neuroscience: beyond the local circuit. Curr. Opin. Neurobiol. 25, xiii–xviii. 10.1016/j.conb.2014.02.002 24602868

[B198] Sotero R. C. Martínez-Cancino R. (2010). Dynamical mean field model of a neural-glial mass. Neural Comput. 22, 969–997. 10.1162/neco.2009.04-09-1002 20028223

[B199] Srinivasan R. Huang B. S. Venugopal S. Johnston A. D. Chai H. Zeng H. (2015). Ca^2+^ signaling in astrocytes from Ip3r2^-/-^ mice in brain slices and during startle responses *in vivo*. Nat. Neurosci. 18, 708–717. 10.1038/nn.4001 25894291 PMC4429056

[B200] Stamatakis M. Mantzaris N. V. (2006). Modeling of ATP-mediated signal transduction and wave propagation in astrocytic cellular networks. J. Theor. Biol. 241, 649–668. 10.1016/j.jtbi.2006.01.002 16460762

[B201] Stamatakis M. Mantzaris N. V. (2007). Astrocyte signaling in the presence of spatial inhomogeneities. Chaos 17:033123. 10.1063/1.2767409 17903005

[B202] Stiles J. R. Bartol T. M. (2001). Monte Carlo methods for simulating realistic synaptic microphysiology using MCell, in Computational Neuroscience: Realistic Modeling for Experimentalists, ed De Schutter E. (Boca Raton, FL: CRC Press), 87–127.

[B203] Suffczynski P. Kalitzin S. Lopes Da Silva F. H. (2004). Dynamics of non-convulsive epileptic phenomena modeled by a bistable neuronal network. Neuroscience 126, 467–484. 10.1016/j.neuroscience.2004.03.014 15207365

[B204] Suzuki A. Stern S. A. Bozdagi O. Huntley G. W. Walker R. H. Magistretti P. J.. (2011). Astrocyte-neuron lactate transport is required for long-term memory formation. Cell 144, 810–823. 10.1016/j.cell.2011.02.018 21376239 PMC3073831

[B205] Taheri M. Handy G. Borisyuk A. White J. A. (2017). Diversity of evoked astrocyte Ca^2+^ dynamics quantified through experimental measurements and mathematical modeling. Front. Syst. Neurosci. 11:79. 10.3389/fnsys.2017.00079 29109680 PMC5660282

[B206] Tang J. Liu T.-B. Ma J. Luo J.-M. Yang X.-Q. (2016). Effect of calcium channel noise in astrocytes on neuronal transmission. Commun. Nonlinear Sci. Numer. Simulat. 32, 262–272. 10.1016/j.cnsns.2015.08.019

[B207] Tang J. Luo J.-M. Ma J. (2013). Information transmission in a neuron-astrocyte coupled model. PLoS ONE 8:e80324. 10.1371/journal.pone.0080324 24312211 PMC3843665

[B208] Tang J. Zhang J. Ma J. Zhang G. Y. Yang X. Q. (2017). Astrocyte calcium wave induces seizure-like behavior in neuron network. Sci. China Tech. Sci. 60, 1011–1018. 10.1007/s11431-016-0293-9

[B209] Tewari S. Majumdar K. (2012a). A mathematical model for astrocytes mediated LTP at single hippocampal synapses. J. Comput. Neurosci. 33, 341–370. 10.1007/s10827-012-0389-5 22454034

[B210] Tewari S. Parpura V. (2013). A possible role of astrocytes in contextual memory retrieval: an analysis obtained using a quantitative framework. Front. Comput. Neurosci. 7:145. 10.3389/fncom.2013.00145 24204341 PMC3817599

[B211] Tewari S. Parpura V. (2014). Data and model tango to aid the understanding of astrocyte-neuron signaling. Front. Comput. Neurosci. 8:3. 10.3389/fncom.2014.00003 24478686 PMC3900764

[B212] Tewari S. G. Majumdar K. K. (2012b). A mathematical model of the tripartite synapse: astrocyte-induced synaptic plasticity. J. Biol. Phys. 38, 465–496. 10.1007/s10867-012-9267-7 23729909 PMC3388198

[B213] Thul R. (2014). Translating intracellular calcium signaling into models. Cold Spring Harbor Protoc. 2014, 463–471. 10.1101/pdb.top066266 24786496

[B214] Tilūnaitė A. Croft W. Russell N. Bellamy T. C. Thul R. (2017). A bayesian approach to modelling heterogeneous calcium responses in cell populations. PLoS Comput. Biol. 13:e1005794. 10.1371/journal.pcbi.1005794 28985235 PMC5646906

[B215] Toivari E. Manninen T. Nahata A. K. Jalonen T. O. Linne M.-L. (2011). Effects of transmitters and amyloid-beta peptide on calcium signals in rat cortical astrocytes: Fura-2AM measurements and stochastic model simulations. PLoS ONE 6:e17914. 10.1371/journal.pone.0017914 21483471 PMC3066169

[B216] Topalidou M. Leblois A. Boraud T. Rougier N. P. (2015). A long journey into reproducible computational neuroscience. Front. Comput. Neurosci. 9:30. 10.3389/fncom.2015.00030 25798104 PMC4350388

[B217] Traub R. D. Wong R. K. Miles R. Michelson H. (1991). A model of a CA3 hippocampal pyramidal neuron incorporating voltage-clamp data on intrinsic conductances. J. Neurophysiol. 66, 635–650. 10.1152/jn.1991.66.2.635 1663538

[B218] Tsodyks M. V. Markram H. (1997). The neural code between neocortical pyramidal neurons depends on neurotransmitter release probability. Proc. Natl. Acad. Sci. U.S.A. 94, 719–723. 10.1073/pnas.94.2.719 9012851 PMC19580

[B219] Ullah G. Jung P. Cornell-Bell A. H. (2006). Anti-phase calcium oscillations in astrocytes via inositol (1,4,5)-trisphosphate regeneration. Cell Calcium 39, 197–208. 10.1016/j.ceca.2005.10.009 16330095

[B220] Valenza G. Pioggia G. Armato A. Ferro M. Scilingo E. P. De Rossi D. (2011). A neuron-astrocyte transistor-like model for neuromorphic dressed neurons. Neural Netw. 24, 679–685. 10.1016/j.neunet.2011.03.013 21441011

[B221] Verkhratsky A. Butt A. M. (2013). Glial Physiology and Pathophysiology. Oxford: John Wiley & Sons.

[B222] Volman V. Bazhenov M. Sejnowski T. J. (2012). Computational models of neuron-astrocyte interaction in epilepsy. Front. Comput. Neurosci. 6:58. 10.3389/fncom.2012.00058 23060780 PMC3459315

[B223] Volman V. Ben-Jacob E. Levine H. (2007). The astrocyte as a gatekeeper of synaptic information transfer. Neural Comput. 19, 303–326. 10.1162/neco.2007.19.2.303 17206866

[B224] Volterra A. Liaudet N. Savtchouk I. (2014). Astrocyte Ca^2+^ signalling: an unexpected complexity. Nat. Rev. Neurosci., 15, 327–335. 10.1038/nrn3725 24739787

[B225] Wade J. McDaid L. Harkin J. Crunelli V. Kelso S. (2012). Self-repair in a bidirectionally coupled astrocyte-neuron (AN) system based on retrograde signaling. Front. Comput. Neurosci. 6:76. 10.3389/fncom.2012.00076 23055965 PMC3458420

[B226] Wade J. McDaid L. Harkin J. Crunelli V. Kelso S. (2013). Biophysically based computational models of astrocyte ~ neuron coupling and their functional significance. Front. Comput. Neurosci. 7:44. 10.3389/fncom.2013.00044 23675340 PMC3646252

[B227] Wade J. J. McDaid L. J. Harkin J. Crunelli V. Kelso J. A. S. (2011). Bidirectional coupling between astrocytes and neurons mediates learning and dynamic coordination in the brain: a multiple modeling approach. PLoS ONE 6:e29445. 10.1371/journal.pone.0029445 22242121 PMC3248449

[B228] Wallach G. Lallouette J. Herzog N. De Pittà M. Jacob E. B. Berry H.. (2014). Glutamate mediated astrocytic filtering of neuronal activity. PLoS Comput. Biol. 10:e1003964. 10.1371/journal.pcbi.1003964 25521344 PMC4270452

[B229] Wang X. Takano T. Nedergaard M. (2009). Astrocytic calcium signaling: mechanism and implications for functional brain imaging, in Dynamic Brain Imaging: Multi-Modal Methods and In Vivo Applications, ed Hyder F. (Totowa, NJ: Humana Press), 93–109. 10.1007/978-1-59745-543-5_5 PMC3638986 18839089

[B230] Wei F. Shuai J. (2011). Intercellular calcium waves in glial cells with bistable dynamics. Phys. Biol. 8:026009. 10.1088/1478-3975/8/2/026009 21378440

[B231] Wils S. De Schutter E. (2009). STEPS: modeling and simulating complex reaction-diffusion systems with Python. Front. Neuroinform. 3:15. 10.3389/neuro.11.015.2009 19623245 PMC2706651

[B232] Witthoft A. Filosa J. A. Karniadakis G. E. (2013). Potassium buffering in the neurovascular unit: models and sensitivity analysis. Biophys. J. 105, 2046–2054. 10.1016/j.bpj.2013.09.012 24209849 PMC3824545

[B233] Witthoft A. Karniadakis G. E. (2012). A bidirectional model for communication in the neurovascular unit. J. Theor. Biol. 311, 80–93. 10.1016/j.jtbi.2012.07.014 22828568

[B234] Yang J. Ruchti E. Petit J.-M. Jourdain P. Grenningloh G. Allaman I.. (2014). Lactate promotes plasticity gene expression by potentiating NMDA signaling in neurons. Proc. Natl. Acad. Sci. U.S.A. 111, 12228–12233. 10.1073/pnas.1322912111 25071212 PMC4143009

[B235] Yang Y. Yeo C. K. (2015). Conceptual network model from sensory neurons to astrocytes of the human nervous system. IEEE Trans. Biomed. Eng. 62, 1843–1852. 10.1109/TBME.2015.2405549 25706505

[B236] Yao W. Huang H. Miura R. M. (2018). Role of astrocyte in cortical spreading depression: a quantitative model of neuron-astrocyte network. Commun. Comput. Phys. 23, 440–458. 10.4208/cicp.OA-2016-0262

[B237] Zeng S. Li B. Zeng S. Chen S. (2009). Simulation of spontaneous Ca^2+^ oscillations in astrocytes mediated by voltage-gated calcium channels. Biophys. J. 97, 2429–2437. 10.1016/j.bpj.2009.08.030 19883585 PMC2770604

